# Supplementary material for: The consequences of exercise-induced weight loss on food reinforcement. A randomized controlled trial
Source: PLoS One. 2020 Jun 18;15(6):e0234692. doi: 10.1371/journal.pone.0234692 (PMC7302707; doi:10.1371/journal.pone.0234692)
Supplement: S1 File — Provided to participants at baseline to ensure they were healthy enough to engage in exercise without physician consent. (PDF) [file pone.0234692.s002.pdf]

Physical Activity Readiness Questionnaire (PAR-Q) and You

Regular physical activity is fun and healthy, and increasingly more people are starting to become more active every day. Being more active is very safe for most people. However, some people should check with their doctor before they start becoming much more physically active.

If you are planning to become much more physically active than you are now, start by answering the seven questions in the box below. If you are between the ages of 15 and 69, the PAR-Q will tell you if you should check with your doctor before you start. If you are over 69 years of age, and you are not used to being very active, check with your doctor.

Common sense is your best guide when you answer these questions. Please read the questions carefully and answer each one honestly:

YES	NO		
☐	☐	1.	Has your doctor ever said that you have a heart condition and that you should only do physical activity recommended by a doctor?
☐	☐	2.	Do you feel pain in your chest when you do physical activity?
☐	☐	3.	In the past month, have you had chest pain when you were not doing physical activity?
☐	☐	4.	Do you lose your balance because of dizziness or do you ever lose consciousness?
☐	☐	5.	Do you have a bone or joint problem that could be made worse by a change in your physical activity?
☐	☐	6.	Is your doctor currently prescribing drugs (for example, water pills) for your blood pressure or heart condition?
☐	☐	7.	Do you know of any other reason why you should not do physical activity?

If you answered:

YES to one or more questions

Talk to your doctor by phone or in person BEFORE you start becoming much more physically active or BEFORE you have a fitness appraisal. Tell your doctor about the PAR-Q and which questions you answered YES.

- You may be able to do any activity you want – as long as you start slowly and build up gradually. Or, you may need to restrict your activities to those which are safe for you. Talk with your doctor about the kinds of activities you wish to participate in and follow his/her advice.
- Find out which community programs are safe and helpful for you.

NO to all questions

If you answered NO honestly to all PAR-Q questions, you can be reasonably sure that you can:

- Start becoming much more physically active – begin slowly and build up gradually. This is the safest and easiest way to go.
- Take part in a fitness appraisal – this is an excellent way to determine your basic fitness so that you can plan the best way for you to live actively.

Delay becoming much more active:

- If you are not feeling well because of a temporary illness such as a cold or a fever – wait until you feel better; or
- If you are or may be pregnant – talk to your doctor before you start becoming more active.

Please note: If your health changes so that you then answer YES to any of the above questions, tell your fitness or health professional. Ask whether you should change your physical activity plan.

Informed use of the PAR-Q: Reprinted from ACSM's Health/Fitness Facility Standards and Guidelines, 1997 by American College of Sports Medicine
