# Supplementary material for: The consequences of exercise-induced weight loss on food reinforcement. A randomized controlled trial
Source: PLoS One. 2020 Jun 18;15(6):e0234692. doi: 10.1371/journal.pone.0234692 (PMC7302707; doi:10.1371/journal.pone.0234692)
Supplement: S2 File — Provided to participants at baseline to ensure the met certain inclusion criteria regarding current or recent health issues. (PDF) [file pone.0234692.s003.pdf]

Health History Questionnaire

NAME: ___________________________ DATE: ___________________________
Birthdate: _______________________________Gender: Male Female
Height:______________________ Weight:_________________ BMI_______

Person to contact in case of Emergency:
Name: ________________________________ Phone: _______________________

1. Are you taking any medications or drugs?
   If YES, please list medication, dose and reason:
   Medication:_________________Dose:_____________Reason:________________
   Medication:_________________Dose:_____________Reason:________________
   Medication:_________________Dose:_____________Reason:________________
   Medication:_________________Dose:_____________Reason:________________
   Use the back for more space

YES	NO	
□	□	1. Has your doctor ever said that you have a heart condition and that you should only do physical activity recommended by a doctor?
□	□	2. Do you feel pain in your chest when you do physical activity?
□	□	3. In the past month, have you had chest pain when you weren't doing physical activity?
□	□	4. Do you lose your balance because of dizziness or do you ever lose consciousness?
□	□	5. Do you have a bone or joint problem that could be made worse by a change in your physical activity?
□	□	6. Is your doctor currently prescribing drugs (for example, water pills) for your blood pressure or heart condition?
□	□	7. Do you know of any other reason why you should not do physical activity?

**General Health Questions:**	**Yes**	**No**
**History of heart problems, chest pain or stroke**		
**Increased blood pressure**		
**History of breathing or lung problems**		
**Diabetes or thyroid disease**		
**Seizures, tremors or severe headaches**		
**Arthritis or other bone problems**		
**Increased blood cholesterol**		
**Use products containing tobacco or nicotine regularly**		
**Recent surgery (last 12 months)**		
**Any other chronic illness or condition Please List:**		
