# Supplementary material for: The consequences of exercise-induced weight loss on food reinforcement. A randomized controlled trial
Source: PLoS One. 2020 Jun 18;15(6):e0234692. doi: 10.1371/journal.pone.0234692 (PMC7302707; doi:10.1371/journal.pone.0234692)
Supplement: S3 File — Scale used to assess hunger and desire to eat prior to each RRV test. (PDF) [file pone.0234692.s004.pdf]

Hunger and Satiety Visual Analogue Scales (VAS)

Date:_______________ Participant #:________________

Please place a vertical line on each of the scales below to illustrate how hungry or full you are right now.

(1) How strong is your desire to eat?

very weak *very strong*

(2) How hungry do you feel?

not hungry at all *as hungry as I have ever felt*

(3) How full do you feel?

not full at all *totally full*

(4) How satisfied do you feel?

completely empty *cannot eat another bite*

(5) How much do you think you could eat right now?

not at all *a large amount*
