# Supplementary material for: The consequences of exercise-induced weight loss on food reinforcement. A randomized controlled trial
Source: PLoS One. 2020 Jun 18;15(6):e0234692. doi: 10.1371/journal.pone.0234692 (PMC7302707; doi:10.1371/journal.pone.0234692)
Supplement: S4 File — Questionnaire used to assess liking of study foods to determine which will be used in the RRV test. (PDF) [file pone.0234692.s005.pdf]

LIKING OF STUDY FOODS

Date________________ Participant #______________________

Please circle the number that best represents how much you like each food listed.

Oreo Cookies

1 2 3 4 5 6 7 8 9 10 11

Do not like it at all *Like it very much*

Mixed Nuts

1 2 3 4 5 6 7 8 9 10 11

Do not like it at all *Like it very much*

Doritos

1 2 3 4 5 6 7 8 9 10 11

Do not like it at all *Like it very much*

Nutri-grain bar

1 2 3 4 5 6 7 8 9 10 11

Do not like it at all *Like it very much*

Dried Banana

1 2 3 4 5 6 7 8 9 10 11

Do not like it at all *Like it very much*

Snickers Bar

1 2 3 4 5 6 7 8 9 10 11

Do not like it at all *Like it very much*
