# Supplementary material for: The consequences of exercise-induced weight loss on food reinforcement. A randomized controlled trial
Source: PLoS One. 2020 Jun 18;15(6):e0234692. doi: 10.1371/journal.pone.0234692 (PMC7302707; doi:10.1371/journal.pone.0234692)
Supplement: S5 File — IRB-approved protocol for present trial. (PDF) [file pone.0234692.s006.pdf]

IRB Approval
9/17/2019
IRB # 45819
CLOSED

PROTOCOL TYPE

Which IRB

(selected) Medical ( ) NonMedical

Protocol Process Type

( ) Exemption
( ) Expedited (Must be risk level 1)
(selected) Full

IMPORTANT NOTE: Once you have saved your choices under "Which IRB" and "Protocol Process Type", you will not be able to change your selections. If you select the wrong IRB Type and/or your application is deemed eligible for a different Protocol Process Type, it may be necessary to create a new application.

Please see below for guidance on which selections to make, and/or go to ORI's "Getting Started" web page. If you still have questions about which IRB or Protocol Process Type to choose, please contact the Office of Research Integrity (ORI) at 859-257-9428 **prior** to saving your selections.

Which IRB

The **Medical IRB** reviews research emanating from the Colleges of Dentistry; Health Sciences; Medicine; Nursing; Pharmacy and Health Sciences; and Public Health.

The **Nonmedical IRB** reviews research originating from the Colleges of Agriculture; Arts & Sciences; Business & Economics; Communications & Information; Design; Education; Engineering; Fine Arts; Law; and Social Work. The Nonmedical IRB does not review studies that involve administration of drugs, testing safety or effectiveness of medical devices, or studies that involve invasive medical procedures, regardless of from what college the application originates.

Which Protocol Process Type

Under federal regulations, an investigator's application to conduct a research project involving human subjects can be processed by the IRBs in three ways:

- by full review;
- by exemption certification;
- by expedited review.

The preliminary determination that a research project is eligible for exemption certification or expedited review is made by the investigator. For assistance in determining which review process type your IRB application is eligible for, please go to ORI's "Getting Started" web page.

The revised Common Rule expanded exemption certification category 4 for certain secondary research with identifiable information or biospecimens. The regulations no longer require the information or biospecimens to be existing. For more information see the Exemption Categories Tool.

CONTINUATION REVIEW/FINAL REVIEW

Based on your responses to the Continuation Review/Final Review questions, to be in accord with federal policy a final review report must be submitted to properly CLOSE OUT your protocol. IF YOU WISH TO EXTEND YOUR IRB APPROVAL PERIOD, update your 'Anticipated Ending Date of Research Project' under the Project Information section and include any other supportive documentation for continuation of your study [NOTE: If you wish for your IRB approval to continue, but you do not request an extension and complete and submit your materials in a timely manner, IRB approval will expire at the end of the current approval period.].

If you have any questions, please contact the Office of Research Integrity at 859-257-9428.

To initiate your continuation review (CR)/annual administrative review (AAR), or properly close your study, complete this section and update/correct all other sections of your IRB application as applicable.
*****IMPORTANT*** Before leaving this page to update other sections of your application, be sure to SAVE this section first.**

1. Status of the Research

Check the statement(s) that best describe(s) the current status of your research:

- [ ] No subjects have enrolled to date.
- [ ] Recruitment and/or enrollment of new subjects or review of records/specimens continue.
- [ ] Study is closed to enrollment, but subjects still receive research-related interventions (e.g., treatment, blood draws).
- [ ] Study enrollment is permanently closed; subjects have completed all research-related interventions; and the study remains active only for long-term follow-up of subjects (see Tool Tip above for info on long-term follow-up of subjects).
- [ ] Research has progressed to the point that it involves 1) Data analysis, including analysis of identifiable private information or identifiable biospecimens; and/or 2) Accessing follow-up clinical data from procedures that subjects would undergo as part of clinical care.
- [ ] The remaining research activities are limited only to data analysis. There is access to records or specimens either directly or through codes or links to the data.*
- [x] The remaining research activities are limited only to data analysis. There is no subject/record/specimen identifying codes or links to the data; the researcher or research team cannot readily ascertain the subject's identity.*
- [ ] All study activities are complete. IRB approval can be inactivated.

*Possibility that review will move from Full to Expedited.

2. If subjects have been enrolled within the last year, and the IRB approved a consent/assent form for your study, attach one copy of the entire signed consent/assent form/HIPAA form for the last TWO subjects enrolled.

Attachments

3. Informed Consent

If the study is **open to subject enrollment, please go to the Informed Consent section of the E-IRB Application and verify attachment(s) include:**

- One clean copy in PDF (without the IRB Approval stamp) of the currently approved consent/assent document(s), or,
- If requesting changes to the consent/assent document(s), submit one copy with the changes highlighted (and designate Document Type as "Highlighted"), and one clean copy in PDF (without the changes highlighted).

If the study is **open to subject enrollment and the IRB has waived the requirement to document informed consent, please go to the Informed Consent section of the E-IRB Application and verify attachment(s) include:**

- One clean copy in PDF of the currently approved document used for the informed consent process (e.g., cover letter, phone script), or,
- If requesting changes to the consent/assent document(s), submit one copy with the changes highlighted (and designate Document Type as "Highlighted"), and one clean copy in PDF (without the changes highlighted).

4. Unanticipated Problems Involving Risk to Subjects or Others/Adverse Events Summary & Assessment

Did any **problems/adverse events** occur during the last 12 months?

(x) Yes ( ) No

In the space below, provide a written summary of both unanticipated problems* and available information regarding adverse events since the last review (e.g., initial review or annual/continuing review). The amount of detail provided in such a summary will vary depending on the type of research being conducted; in many cases, such a summary could be a brief statement that there have been no unanticipated problems and that adverse events have occurred at the expected frequency and level of severity as documented in the research protocol, the informed consent document, and investigator's brochure (if applicable). **The summary must include the PI's assessment whether the problems/adverse events warrant changes to the protocol, consent process, or risk/benefit ratio.**

Note: It is the IRB's expectation that all unanticipated problems involving risk to subjects or others or related deaths requiring prompt reporting are submitted in the appropriate time frame (See Policy [PDF]). Your response to this Annual/Continuing Review is considered assurance that all prompt reportable problems/adverse events have been submitted for IRB review.

An old version (2017-2018) consent form was accidentally used for a few participants in 2019. There was no difference in the consent forms.

*For multisite studies, the written summary should describe external events determined to be unanticipated problems involving risk to subjects or others.

5. Subject Info To-Date

Our records for the previously approved IRB application indicate the **IRB approved estimate** of subjects to be enrolled (or records/specimens reviewed) is:

60

Enter the number of enrolled subjects (or records/specimens reviewed) that **have not been previously reported** to the IRB

80

Our records for the previously approved IRB application indicate the previous total # of subjects enrolled (or records/specimens reviewed) since activation of the study is:

0

The new total number of subjects enrolled (or records/specimens reviewed) since activation of the study:

80

Please review the Project Info section for the IRB approved estimate of subjects to be enrolled (or records/specimens reviewed). If this new total exceeds subjects to be enrolled (or records/specimens reviewed), please update the number in the field for Number of Human Subjects in the Project Info section.

6. Data and Safety Monitoring Board (DSMB)

Our records for the previously approved IRB application indicate:
This **study is monitored** by a Data and Safety Monitoring Board (DSMB): **N**

There is a Data and Safety Monitoring Plan: **Y**

If yes, to any of the above, attach all documentation (i.e. summary report; meeting minutes) representing Data and Safety Monitoring activities that have not been previously reported to the IRB.

Attachments

7. Since the most recent IRB Initial/Continuation Review Approval:

Have there been any **participant complaints** regarding the research?

○ Yes ● No

If yes, in the field below, provide a summary describing the complaints.

Have any **subjects withdrawn** from the research?

● Yes ○ No

If yes, in the field below, provide a detailed explanation.

one due to injury, one due to sickness, one got pregnant.

Has any **new and relevant literature** been published since the last IRB review, especially literature relating to risks associated with the research?

○ Yes ● No

If yes, attach a copy of the literature as well as a brief summary of the literature including, if pertinent, the impact of the findings on the protection of human subjects.

Attachments

Have there been any **interim findings?**

● Yes ○ No

If yes, attach a copy of **Interim Findings**.

Attachments

Have **subjects experienced any benefits?**

● Yes ○ No

If yes, in the field below, provide a description of benefits subjects have experienced.

Many have lost significant amount of weight.

Have there been any **inspections/audits/quality improvement reviews** of your research protocol resulting in the need for corrective action in order to protect the safety and welfare of subjects?

○ Yes ● No

If yes, please attach documentation evidencing the outcome(s) and any corrective action(s) taken as a result.

Attachments

Was an FDA 483 issued as a result of any inspections/audits?

○ Yes ● No

If yes, submit documentation using attachment button above.

8. Risk Level:

Our records for the previously approved IRB application show your research is:

Risk Level: 3

Has something during the course of your research changed the level of risk?

○ Yes ● No

If yes, go to the Risk Level section, mark the appropriate risk level, and in the field below, describe why the risk level has changed:

9. Funding/Support:

Our records for the **previously approved** IRB application indicate your research is being submitted to, supported by, or conducted in cooperation with the following external or internal agency(ies) or funding program(s):

- ☐ Grant application pending
- ☐ (HHS) Dept. of Health & Human Services
  - ☐ (NIH) National Institutes of Health
  - ☐ (CDC) Centers for Disease Control & Prevention

- (HRSA) Health Resources and Services Administration
- (SAMHSA) Substance Abuse and Mental Health Services Administration
- (DoJ) Department of Justice or Bureau of Prisons
- (DoE) Department of Energy
- (EPA) Environmental Protection Agency
- Federal Agencies Other Than Those Listed Here
- Industry (Other than Pharmaceutical Companies)
- Internal Grant Program w/ proposal
- Internal Grant Program w/o proposal
- National Science Foundation
- Other Institutions of Higher Education
- Pharmaceutical Company
- Private Foundation/Association
- U.S. Department of Education
- State

Other:

department start up funds

Please **update the Funding/Support section of your IRB application** if needed, including the following attachments if they contain changes not previously reported to the IRB:

- A current copy of your **protocol if you are conducting industry/pharmaceutical research;**
- A current **Investigator Brochure** (submit a copy with all changes underlined).
- A **new or revised grant application** for this project.

Did your project receive extramural funding?

○ Yes ◉ No

If yes, please review and correct if necessary, the OSPA Account # information under the **Funding/Support section** of your IRB application.

If the project is externally funded, has the sponsor offered any of the research team enrollment incentives or other personal benefit bonuses? (e.g., cash/check, travel reimbursements, gift checks, etc.)

○ Yes ○ No ◉ N/A

Note: It is University of Kentucky policy that personal benefit bonuses are not allowed. If these conditions change during the course of the study, please notify the IRB.

10. Project Information

Our records for the previously approved IRB application indicate your estimated project end date is:

12/01/2019

If you have a new estimated project end date, please go to the Project Info section and change the date in the field for Anticipated Ending Date of Research Project.

11. Study Personnel

Our records for the previously approved IRB application indicate the following individuals are study personnel on this project (if applicable):

Last Name	First Name
Holbrook	Kathryn
Rice	Linda
Chamblin	Lisa
Tillery	Melanie
True	Laura
Long	Douglas
Black	William
Daniel	Stephanie
Mason	Jalyn
Stiehler	Julie
Moreland	Jack
Hays II	Harry

Please review the individuals listed above and update your records as needed in the Study Personnel section of the E-IRB application, being sure that each individual listed has completed or is up-to-date on the mandatory human research protection training [see the policy on Mandatory Human Subject Protection Training FAQs (required every three years)].

12. Progress of the Research

To meet federal requirements the IRB is relying on your RESEARCH DESCRIPTION as a protocol summary and their expectation is that it is up-to-date. If the currently approved protocol (or research description) in your E-IRB application is outdated, please make applicable changes, and describe in the field below any substantive changes and explain why they are essential. If none, insert "N/A" in the text field below. If you are closing your study, you may use the space below to summarize the final status of the research.

currently up to date

Note: No changes in the research procedures should have occurred without previous IRB review. Approval from the IRB must be obtained before implementing any changes.

Provide a brief **summary** of any **modifications that affect subject safety and/or welfare** approved by the IRB since the last initial or continuation review (If none, insert "N/A" in the text field below.):

no modifications

If you are conducting the study under your own IND/IDE (Investigator Sponsored), attach one copy of the most recent progress report sent to the FDA.

Attachments

13. Confidentiality/Security

Review your Research Description section and update the Confidentiality portion, if necessary, to describe measures for security of electronic and physical research records (e.g., informed consent document(s), HIPAA Authorization forms, sensitive or private data).

14. Subject Demographics

Our records for the previously approved IRB application indicate the following categories of subjects and controls are included in your research:

- [ ] Children (individuals under age 18)
- [ ] Wards of the State (Children)
- [ ] Emancipated Minors
- [ ] Students
- [ ] College of Medicine Students
- [ ] UK Medical Center Residents or House Officers
- [ ] Impaired Consent Capacity Adults
- [ ] Pregnant Women/Neonates/Fetal Material
- [ ] Prisoners
- [ ] Non-English Speaking
- [ ] International Citizens
- [x] Normal Volunteers
- [ ] Military Personnel and/or DoD Civilian Employees
- [ ] Patients
- [ ] Appalachian Population

Please review the Subject Demographics section of your IRB application for accuracy, and note the following:

If during the course of your research 1) any prisoners have been enrolled, OR 2) subjects have been enrolled that became involuntarily confined/detained in a penal institution that have not been previously reported to the IRB, go to Subject Demographic section in your E-IRB application and mark “prisoners” in the categories of subjects to be included in the study, if it is not already marked.

Note: If either 1 or 2 above apply, and you have received funding from the Department of Health and Human Services (HHS), a Certification Letter should have been submitted to the Office for Human Research Protections (OHRP); prisoners and individuals who have become involuntarily confined/detained in a penal institution cannot continue participation in the research until OHRP issues approval. If the Certification has not been submitted, contact the Office of Research Integrity.

Based on the **total # of subjects** who have enrolled, complete the subject demographic section below:

Ethnic Origin	#Male	#Female
American Indian/Alaskan Native:	0	0
Asian:	0	1
Black/African American:	2	3
Hispanic/Latino:	1	2
Native Hawaiian/Pacific Islander:		
White/Caucasian:	15	56
Other or Unknown:		

If unknown, please explain why:

15. Research Sites

Our records for the previously approved IRB application indicate that you are conducting research at the following sites:

UK Sites

- [x] UK Classroom(s)/Lab(s)
- [ ] UK Clinics in Lexington
- [ ] UK Clinics outside of Lexington
- [ ] UK Healthcare Good Samaritan Hospital
- [x] UK Hospital

Schools/Education Institutions Schools/Education Institutions

- [ ] Fayette Co. School Systems *
- [ ] Other State/Regional School Systems

- [ ] Institutions of Higher Education (other than UK)

Other Medical Facilities

- [ ] Bluegrass Regional Mental Health Retardation Board
- [ ] Cardinal Hill Hospital
- [ ] Eastern State Hospital
- [ ] Nursing Homes
- [ ] Shriner's Children's Hospital
- [ ] Other Hospitals and Med. Centers

- [ ] Correctional Facilities
- [ ] Home Health Agencies
- [ ] International Sites

Other:

If the above listed sites are not accurate, go to the Research Sites section of the E-IRB application to update the facilities at which research procedures have been or will be conducted.

If you are adding a new off-site facility, you may also need to update your E-IRB application Research Description, Research Sites, Informed Consent, and other affected sections as well as any documents which will list the off-site facility. Documents needing updating may include, but not limited to:

- Consent forms (attachment under Informed Consent section)
- Brochures (attachment under Additional Info section)
- Advertisements (attachment under Research Description section) ;
- Letter of support (attachment under Research Sites section)).

Please revise applicable sections and attachments as necessary.

16. Disclosure of Significant Financial Interest

Disclosure of Significant Financial Interest:

Our records for the previously approved IRB application indicate that you, your investigators, and/or key personnel (KP) have a significant financial interest (SFI) related to your/their responsibilities at the University of Kentucky (that requires disclosure per the UK administrative regulation 7:2):

○ Yes ● No

If you need to update your records, please go to the PI Contact Information section and/or Details for individuals listed in the Study Personnel section to change your response to the applicable question(s).

17. Supplementals

To ensure the IRB has the most accurate information for your protocol you are expected to re-visit the E-IRB application sections and make corrections or updates as needed. At a minimum you are being asked to review the following sections for accuracy:

STUDY DRUG INFORMATION—Please review for accuracy.
STUDY DEVICE INFORMATION—Please review for accuracy.
RESEARCH ATTRIBUTES—Please review for accuracy.
OTHER REVIEW COMMITTEES -- Please review for accuracy.

If applicable, submit one copy of the entire **signed HIPAA Authorization form** for the same last TWO subjects enrolled.

Attachments

Attach Type	File Name
CR_EntireHIPAAAuthorization	consents.pdf

PROJECT INFORMATION

Title of Project: (If applicable, use the exact title listed in the grant/contract application).

Exercise and Weight Control: A Search for Biological and Neurobehavioral Compensatory Mechanisms that Defend Against Exercise-Induced Negative Energy Balance

Short Title Description
Note: "Short Title" should consist of a couple key words to easily identify your study - these key words (rather than the whole title) will be displayed on the Dashboard in the listing for your study.

Exercise and Weight Control: A Search for Biologic

Anticipated Ending Date of Research Project: 12/1/2019

Number of human subjects 60

Study is/will be open to new subject enrollment or data/specimen collection: ○ Yes ◉ No

PI CONTACT INFORMATION

The Principal Investigator's (PI) contact information is filled in automatically based on who was logged in when the application was created (with LinkBlue ID). If research is being submitted to or supported by an extramural funding agency such as NIH, a private foundation or a pharmaceutical/manufacturing company, the PI listed on the grant application or the drug protocol must be the same person listed below.

If you are not the Principal Investigator, do NOT add yourself as study personnel. You may change the PI contact information on an application that is in Researcher edit mode by:

- clicking the "Change Principal Investigator" link below;
- searching for the PI's name using the search feature;
- clicking "Select" by the name of the Principal Investigator, then "Save Contact Information".

You will automatically be added as study personnel with edit authorization so you can continue editing the application.

Please fill in any blank fields with the appropriate contact information (gray shaded fields are not editable). Required fields left blank will be highlighted in pink after you click "Save".

To change home and work addresses, go to myUK and update using the Employee Self Service (ESS) portal. If name has changed, the individual with the name change will need to submit a 'Name Change Form' to the Human Resources Benefits Office for entering into SAP. The new name will need to be associated with the individual's Link Blue ID in SAP before the change is reflected in E-IRB. Contact the HR Benefits Office for additional information.

Note: Principal Investigator (PI) role for E-IRB access
The PI is the individual holding primary responsibility on the research project with the following permissions on the E-IRB application:

1. Read;
2. write/edit;
3. receive communications; and
4. submit to the IRB (IR, CR, MR, Other Review*).

Change Principal Investigator:

First Name:	Kyle	Room# & Bldg:	203 Funkhouser Bldg
Last Name:	Flack	Speed Sort#:	405060054
Middle Name	D.		
Department:	Dietetics and Human Nutrition…	Dept Code:	81500
PI's Employee/Student ID#:	12299414	Rank:	Assistant Professor
PI's Telephone #:	859-257-4351	Degree:	PhD, RD
PI's e-mail address:	kyle.flack@uky.edu	PI's FAX Number:	
PI is R.N.	○ Yes ◉ No	Trained:	Yes
		Date Trained	9/7/2017

Do you, the PI, have a significant financial interest related to your responsibilities at the University of Kentucky (that requires disclosure per the UK administrative regulation 7:2)?

○ Yes ◉ No

RISK LEVEL

Indicate which of the categories listed below accurately describes this protocol

- ○ (Risk Level 1) Not greater than minimal risk
- ○ (Risk Level 2) Greater than minimal risk, but presenting the prospect of direct benefit to individuals subjects
- ◉ (Risk Level 3) Greater then minimal risk, no prospect of direct benefit to individual subjects, but likely to yield generalizable knowledge about the subject's disorder or condition.
- ○ (Risk Level 4) Research not otherwise approvable which presents an opportunity to understand, prevent, or alleviate a serious problem affecting the health or welfare of subjects.

*"Minimal risk" means that the probability and magnitude of harm or discomfort anticipated in the research are not greater in and of themselves from those ordinarily encountered in daily life or during the performance of routine physical or psychological examination or tests [45 CFR 46.102(i)]

Download UK's guidance document on assessing the research risk for additional information on risk [PDF]

SUBJECT DEMOGRAPHICS

Age level of human subjects: (i.e., 6 mths.; 2yrs., etc..) 18 to 40

Indicate the targeted/planned enrollment of the following members of minority groups and their subpopulations
(Please note: The IRB will expect this information to be reported at Continuation Review time):

Enter Numbers Only!

Ethnic Origin	#Male	#Female
American Indian/Alaskan Native:		
Asian:	2	5
Black/African American:	3	5
Hispanic/Latino:	2	5
Native Hawaiian/Pacific Islander:		
White/Caucasian:	11	30
Other or Unknown:		

If unknown, please explain why:

Indicate the categories of subjects and controls to be included in the study. Depending on the subject category applicable to your research you may be required to complete additional forms. [Note, if the study does not involve direct intervention or direct interaction with subjects, (e.g., record-review research, outcomes registries), do not check mark populations which the research does not specifically target. For instance, a large record review of a diverse population may incidentally include a prisoner or an international citizen, but, if the focus or intent of the study has nothing to do with that status, you do not need to check those category(ies).]

Check All That Apply (at least one item must be selected)

- ☐ Children (individuals under age 18)
- ☐ Wards of the State (Children)
- ☐ Emancipated Minors
- ☐ Students
- ☐ College of Medicine Students
- ☐ UK Medical Center Residents or House Officers
- ☐ Impaired Consent Capacity Adults
- ☐ Pregnant Women/Neonates/Fetal

ADDITIONAL INFORMATION:

Please visit the IRB Survival Handbook under the named topic:

- Children/Emancipated Minors
- Students as Subjects
- Prisoners
- Impaired Consent Capacity Adults: Link to required Form

And/Or:

- UKMC Residents or House Officers [see requirement of GME]

45819
CLOSED

Material<br>☐ Prisoners<br>☐ Non-English Speaking<br>☐ International Citizens<br>☑ Normal Volunteers<br>☐ Military Personnel and/or DoD Civilian Employees<br>☐ Patients<br>☐ Appalachian Population	• Non-English Speaking [see instructions for recruitment and E-IRB Research Description section on same topic]<br><br>• International Citizens [HTML] (DoD SOP may apply [PDF])<br><br>• Military Personnel and/or DoD Civilian Employees (DoD SOP may apply [PDF])

The next questions involve assessment of the study relative to potential recruitment of subjects with impaired consent capacity (or likelihood).

☐ Check this box if your study does not involve direct intervention or direct interaction with subjects (e.g., record-review research, secondary data analysis). (you will not need to answer the impaired consent capacity questions)

Does this study focus on adult subjects with any of the clinical conditions listed below that present a high *likelihood* of impaired consent capacity or *fluctuations* in consent capacity? (see examples below)

○ Yes ● No

If Yes, go to the following link and complete and attach the indicated form unless you are filing for an exemption certification: https://ris.uky.edu/ori/oriforms/formt/Scale.asp

Examples of such conditions include:

- Traumatic brain injury or acquired brain injury
- Severe depressive disorders or Bipolar disorders
- Schizophrenia or other mental disorders that
  involve serious cognitive disturbances
- Stroke
- Developmental disabilities
- Degenerative dementias
- CNS cancers and other cancers with possible CNS involvement
- Late stage Parkinson’s Disease
- Late stage persistent substance dependence
- Ischemic heart disease
- HIV/AIDS
- COPD
- Renal insufficiency
- Diabetes
- Autoimmune or inflammatory disorders
- Chronic non-malignant pain disorders
- Drug effects
- Other acute medical crises

Attachments

INFORMED CONSENT/ASSENT PROCESS/WAIVER

For your informed consent attachment(s), please download the most up-to-date version listed in "All Templates" under the APPLICATION LINKS menu on the left, and revise to be in accord with your research project.

Additional Resources:

- Sample Repository/Registry/Bank Consent (PDF) (Word)
- Instructions for Proposed Informed Consent Document
- Instructions for Proposed Assent Form

Consent/Assent Tips:

- **If you have multiple consent documents, be sure to upload each individually (not all in a combined file).**
- **Changes to consent documents (e.g., informed consent form, assent form, cover letter, etc...) should be reflected in a 'tracked changes' version and uploaded separately with the Document Type "Highlighted Changes".**
- **It is very important that only the documents you wish to have approved by the IRB are attached; DELETE OUTDATED FILES -- previously *approved* versions will still be available in Protocol History.**
- **Attachments that are assigned a Document Type to which an IRB approval stamp applies will be considered the version(s) to be used for enrolling subjects once IRB approval has been issued.**
  Document Types that do NOT get an IRB approval stamp are:
  - **"Highlighted Changes",**
  - **"Phone Script", and**
  - **"Sponsor's Sample Consent Form".**

How to Get the Informed Consent Section Check Mark

1. **You must check the box for at least one of the consent items and/or check mark one of the waivers, then if applicable attach the corresponding document(s) as a PDF (if open to enrollment).**
2. **If you no longer need a consent document approved (e.g., closed to enrollment), or, the consent document submitted does not need a stamp for enrolling subjects (e.g., umbrella study, or sub-study), only check mark the "Stamped Consent Doc(s) Not Needed".**
3. **After making your selection(s) be sure to scroll to the bottom of this section and SAVE your work!**

Check All That Apply

- [x] Informed Consent Form (and/or Parental Permission Form)
- [ ] Assent Form
- [ ] Cover Letter (for survey/questionnaire research)
- [ ] Phone Script
- [ ] Informed Consent/HIPAA Combined Form
- [ ] Debriefing and/or Permission to Use Data Form
- [ ] Sponsor's sample consent form for Dept. of Health and Human Services (DHHS)-approved protocol
- [ ] Stamped Consent Doc(s) Not Needed

Attachments

- [ ] Request for Waiver of Informed Consent Process

If you are requesting IRB approval for waiver of the requirement for the informed consent process, or alteration of some or all of the elements of informed consent (i.e. medical record review, deception research, or collection of biological specimens), complete Section 1 and Section 2 below.

Note: The IRB does not approve waiver or alteration of the consent process for greater than minimal risk research, except for planned emergency/acute care research as provided under FDA regulations. Contact ORI for regulations that apply to single emergency use waiver or acute care research waiver (859-257-9428).

SECTION 1.

Check the appropriate item:

☐ I am requesting waiver of the requirement for the informed consent process.

☐ I am requesting alteration of the informed consent process.

If you checked the box for this item, describe which elements of consent will be altered, and/or omitted, and justify the alteration.

SECTION 2.

The IRB may consider your request provided that **all** of the following conditions apply to your research and are appropriately justified. Explain in the space provided for each condition how it applies to your research.

a) The research involves no more than minimal risk to the subject.

b) The rights and welfare of subjects will not be adversely affected.

c) The research could not practicably be carried out without the requested waiver or alteration.

d) Whenever possible, the subjects or legally authorized representatives will be provided with additional pertinent information after they have participated in the study.

45819
CLOSED

If you are requesting IRB approval for waiver of the requirement for documentation of informed consent (i.e. telephone survey or mailed survey, internet research, or certain international research), **your research activities must fit into one of three regulatory options**:

1. The only record linking the participant and the research would be the consent document, and the principal risk would be potential harm resulting from a breach of confidentiality (i.e., a study that involves participants who use illegal drugs).
2. The research presents no more than minimal risk to the participant and involves no procedures for which written consent is normally required outside of the research context (i.e. a cover letter on a survey, or a phone script).
3. The participant (or legally authorized representative) is a member of a distinct cultural group or community in which signing forms is not the norm, and the research presents no more than minimal risk to the subject and there is an appropriate alternative mechanism for documenting that informed consent was obtained.

Select the option below that best fits your study, and explain in the space provided how your study meets the criteria for the selected regulatory option.

Note: The IRB cannot waive the requirement for documentation or alter the consent form for FDA-regulated research unless it meets Option #2 below. FDA does not accept Option #1.

Note: Even if a waiver of the requirement for documentation is approved by the IRB, participants must still be provided oral or written (e.g., cover letter) information including all required and appropriate elements of consent so they have the knowledge and opportunity to consider whether or not to participate. To help ensure required elements are included in your consent document, please use the **Cover Letter Template** as a guide: *English*- [WORD], Spanish- [WORD] The cover letter template was developed specifically for survey/questionnaire research; however, it may be useful as a guide for developing a consent document for other types of research as well.

Option 1

a) The only record linking the participant and the research would be the consent document:

b) The principal risk would be potential harm resulting from a breach of confidentiality (i.e., a study that involves subjects who use illegal drugs).

Under this option, each participant (or legally authorized representative) must be asked whether (s)he wants to sign a consent document; if the participant agrees to sign a consent document, only an IRB approved version should be used.

Option 2

a) The research presents no more than minimal risk to the participant:

b) Involves no procedures for which written consent is normally required outside of the research context (i.e. a cover letter on a survey, or a phone script):

Option 3

a) The subject (or legally authorized representative) is a member of a distinct cultural group or community in which signing forms is not the norm.

b) The research presents no more than minimal risk to the subject.

c) There is an appropriate alternative mechanism for documenting that informed consent was obtained.

STUDY PERSONNEL

Do you have study personnel who will be assisting with the research?
After selecting 'Yes' or 'No' you must save by hitting the 'Save Study Personnel Information' button.

(•) Yes ( ) No

Manage Study Personnel

Identify other study personnel assisting in research project:

- The individual listed as PI in the 'PI Contact Information' section should NOT be added to this section.
- If the research is being completed to meet the requirements of a University of Kentucky academic program, the faculty advisor is also considered study personnel and should be listed as such below.
- Role: DP = Editor (individual can view, navigate, and edit the application for any review phase (IR, CR, MR) or 'Other Review", and submit Other Reviews on behalf of the PI.)
- Role: SP = Reader (individual can view and navigate through the currently approved application only.)

To add an individual via the below feature, search for applicable personnel first, then click "select" by the listing for the person you want to add as study personnel to your protocol. For each individual selected, be sure to specify responsibility in the project, whether authorized by the principal investigator to obtain informed consent, AND denote who should regularly receive E-IRB notifications.

NOTE: Study personnel are required to receive human research protection (HSP) training before implementing any research procedures (e.g., CITI). For information about mandatory training requirements for study personnel, visit UK's FAQ's on Mandatory Training web page, or contact ORI at 859-257-9428. If you have documentation of current HSP training other than that acquired through UK CITI, you may submit it to ORI (Jen.Hill@uky.edu) for credit.

Study personnel assisting in research project:

Last Name	First Name	Responsibility In Project	Role	A C	Contact	Degree	StatusFlag	(HSP)	(HSP)Date	Removed?	Last Updated	SFI
Black	William	Medical Supervisor	SP	N	N	MD	P	Y	06/18/2018	N	07/10/2018	N
Chamblin	Lisa	Data Collection	SP	N	N		P	Y	08/05/2019	N	07/10/2018	N
Daniel	Stephanie	Data Collection	SP	Y	N		P	Y	11/28/2017	N	07/10/2018	N
Hays II	Harry	Data Collection	SP	Y	N	BA	P	Y	11/17/2017	N	07/10/2018	N
Holbrook	Kathryn	Data Collection	SP	N	N		P	Y	12/12/2018	N	07/10/2018	N
Long	Douglas	Data Collection	SP	N	N		P	Y	05/06/2019	N	07/10/2018	N
Mason	Jalyn	Data Analysis/Processing	SP	Y	N		P	Y	06/11/2018	N	07/10/2018	N
Moreland	Jack	Data Collection	SP	Y	N		P	Y	11/17/2017	N	07/10/2018	N
Rice	Linda	Data Collection	SP	N	N		P	Y	10/04/2018	N	07/10/2018	N
Stiehler	Julie	Data Collection	SP	N	N		P	Y	06/11/2018	N	07/10/2018	N
Tillery	Melanie	Data Collection	SP	N	N		P	Y	01/10/2019	N	07/10/2018	N
True	Laura	Data Collection	SP	N	N	RN	P	Y	01/02/2019	N	04/25/2019	N

RESEARCH DESCRIPTION

****!!!PLEASE READ!!!** Known Issue: The below text boxes do not allow symbols, web addresses, or special characters (characters on a standard keyboard should be ok). If something is entered that the text boxes don't allow, user will lose unsaved information.**

Workaround(s):

- **Save your work often to avoid losing data.**
- **Use one of the attachment buttons in this section, or under the Additional Information section to include the information with your application. During the document upload process, you will be able to provide a brief description of the attachment.**

Background: Provide an introduction and background information. Describe past experimental and/or clinical findings leading to the formulation of your study. For research involving investigational drugs, describe the previously conducted animal and human studies. You may reference grant application/sponsor's relevant protocol pages and attach as an appendix in the E-IRB "Additional Information" section, however, a summary paragraph must be provided in the text box below. For research that involves FDA approved drugs or devices, describe the FDA approved uses of this drug/device in relation to your protocol. Attach a copy of the approved labeling as a product package insert or from the Physician's Desk Reference in the applicable E-IRB "Study Drug" or "Study Device" section.

Most people have an enormous capacity to increase their energy expenditure to promote a negative energy balance. Depending on an individual's aerobic fitness, exercise intensities can be maintained for prolonged periods at 2- to 16-fold above resting rates of energy expenditure. As such, 250 to 2500 kcal can be expended during a single exercise session resulting in an acute energy deficit that can be repeated across days. Moreover, exercise promotes the health of almost every organ system and psychological health [1-3]. Yet, exercise without reductions in energy intake is not always effective for weight loss. Potential challenges for using exercise to maintain energy balance include compensatory mechanisms that resist maintenance of negative energy balance by increasing the reinforcing (motivating) value of food (RRVfood), or increasing appetite, which would both promote greater energy intake. A series of satiety signals (e.g.; peptide YY, GLP-1, leptin, pancreatic polypeptide and ghrelin) regulate energy balance and influence hunger, satiety and eating behavior [4, 5]. Many of these peptides exert their effects at the hypothalamic appetite-control center and mesoaccumbal reward areas of the brain, which is essential for experiencing reward from eating food and many other behaviors [6]. An increased motivation for eating caused by exercise-induced energy deficits may reflect an alteration in the balance of control such that the appetitive reward system becomes dominate over satiety signals and the hypothalamic control system [7]. Exercise training alters the secretion of many hormones that could influence hunger, satiety, food reinforcement and energy balance [5, 8-10]. Although many studies have shown suppression of the orexigenic hormone ghrelin and increased concentration of anorexigenic hormones after an acute bout of exercise [11], long-term exercise programs to induce weight loss may have differing effects [12]. Understanding how to increase exercise in a manner that minimizes biological compensatory responses that promote an increase in RRVfood and hunger may be fundamental to utilizing exercise as a means of weight control.
Exercise reinforcement (RRVexercise) influences the choice to be physically active rather than engage in sedentary behavior [13, 14]. RRVexercise predicts the choice to engage in physical activity at a frequency sufficient to meet weekly physical activity guidelines [15] and is a better predictor of exercise behaviors than liking [16]. Thus, increasing RRVexercise could shift choice towards physical activity and away from more reinforcing yet less healthy sedentary alternatives. One way to increase behavioral reinforcement may be through "incentive sensitization" [17]. Incentive Sensitization Theory posits that the RRV of a behavior is increased through repeated exposures, which produce neuroadaptations that increase craving of the behavior [18]. A 'sensitization' or hypersensitivity to the incentive motivational effects of the stimulus follows after repeated exposures to the stimulus. The result is a bias of attentional processing towards the stimuli (i.e., increases its salience within the environment; incentive salience) [19, 20] producing an increase in RRV of the stimulus relative to a competing alternative. Another factor that may be associated with exercise reinforcement is the tolerance to the unpleasant aspects of exercise [15, 21, 22]. Unpleasant aspects of intense exercise such as muscle pain, fatigue, and breathing hard can elicit lower ratings of pleasure and affect [15, 21, 22]. This is especially observed in novice exercisers and overweight individuals [23], which may make it difficult for these individuals to adhere to an exercise program. Indeed, individuals who have greater tolerance for these unpleasant aspects of exercise are more likely to meet PA guidelines [15], pointing to the possibility that the ability to tolerate the unpleasant aspects of exercise is important in adhering to an exercise program.

The proposed research will advance our understanding of how exercise could be used most effectively to promote weight control. The research will identify if some individuals are more likely to lose weight via exercise based on their biological and neurobehavioral compensatory responses to exercise-induced energy deficits. Recent research suggests that exercise at 3,000 kcal/week is sufficient to induce compensatory responses and significant weight loss [24]. However these studies used a 5-6 day/week exercise program. Less is known about the frequency of exercise and its impact on compensatory responses. The ability to achieve a greater energy deficit due to a smaller compensatory response with exercise would have enormous implications for exercise prescription and to the overweight/obese populations.

Secondly, the proposed research will further investigate if incentive sensitization can occur for exercise, if tolerance to exercise intensity plays a role in RRVexercise, and if exercise frequency impacts these variables. Follow up measures will be performed to determine if exercise participation and weight loss can be maintained among previously sedentary individuals following the exercise intervention.

The major innovations will be to concurrently study biological and neurobehavioral mechanisms of compensation and exercise participation at two levels of exercise frequency. Study of the behavioral mechanisms is particularly novel and will include individual

differences in changes in RRVfood, RRVexercise, and the development of tolerance for the aversive properties context of a 12-week exercise intervention.

Objectives: List your research objectives. You may reference grant application/sponsor's relevant protocol pages and attach as an appendix in the E-IRB "Additional Information" section, however, a summary paragraph must be provided in the text box below.

2. Objectives: The objective of this project is to determine the biological, neurobehavioral, and behavioral compensatory responses to exercise training-induced energy deficits in overweight to obese men and women. Specific hypotheses include:

H1: (Primary) Accumulated negative energy balance will be greater when exercising 2 times per week than exercising 6 times per week at 3000 kcal/week for 12 weeks.
H2: The RRVfood will increase more in response to 6 weekly exercise sessions than 2 sessions after 12 weeks of exercise.
H3: Energy intake will increase more in response to 6 weekly exercise sessions than 2 sessions after 12 weeks.
H4: Changes in GLP-1 concentration and RRVfood will be positively associated.
H5: GLP-1, PYY, CCK, leptin, and pancreatic polypeptide concentrations will decrease in response to a meal more with 6 weekly exercise sessions than 2 sessions.
H6: Ghrelin concentrations will increase in response to a meal more with 6 weekly exercise sessions than 2 sessions.
H7: RRVexercise will increase more in response to 2 weekly exercise sessions than 6 sessions after 12 weeks.
H8: Tolerance to exercise discomfort will increase more in response to 2 weekly exercise sessions than 6 sessions after 12 weeks.
H9: The RRVexercise and tolerance to exercise discomfort will be positively associated with maintenance of exercise behaviors 4 weeks after the intervention (week 16).
H10: Changes in hunger hormones/peptides after 12 weeks of exercise will predict weight loss maintance at week 17.

Study Design: Describe the study design (e.g., single/double blind, parallel, crossover, etc.). Indicate whether or not the subjects will receive placebo medication at some point in the research procedures. Also, indicate whether or not the subjects will be randomized in this study. You may reference sponsor's protocol pages and attach as an appendix in the E-IRB "Additional Information" section, however, a summary paragraph must be provided in the text box below. (Including the study design table from a sponsor's protocol is helpful to IRB members.)

Community-Based Participatory Research: If you are conducting community-based participatory research (CBPR), describe strategies for involvement of community members in the design and implementation of the study, and dissemination of results from the study.

Research Repositories: If the purpose of this submission is to establish a Research Repository (bank, registry) indicate whether the material you plan to collect would or would not be available from a commercial supplier, clinical lab, or established IRB approved research repository. Provide scientific justification for establishment of an additional repository collecting duplicate material. Describe the repository design and operating procedures. For relevant information to include, see the UK Research Biospecimen Bank Guidance [PDF] or the UK Research Registry Guidance [PDF]

A factorial design will be employed with exercise frequency (6 days/week or 2 days/week) treated as a between-subjects variable and time (baseline, end of training, end follow-up) treated as a within subjects variable. Subjects will be randomly assigned to exercise groups (6 days/week, 2 days/week) or sedentary control. The control group will be offered the intervention (personalized exercise plan) after follow-up measures.

Attachments

Back to Top

Study Population: Describe the characteristics of the subject population, such as anticipated number, age range, gender, ethnic background and health status. Identify the criteria for inclusion and exclusion. Explain the rationale for the use of special classes such as fetuses, pregnant women, children, institutionalized, adults with impaired consent capacity, prisoners, economically or educationally disadvantaged persons or others who are likely to be vulnerable.

If women or minorities are included, please address how the inclusion of women and members of minority groups and their subpopulations will help you meet your scientific objectives. Exclusion of women or minorities requires clear and compelling rationale that shows inclusion is inappropriate with respect to the health of the subjects or that inclusion is inappropriate for the purpose of the study. Cost is not an acceptable reason for exclusion except when the study would duplicate data from other sources. Women of childbearing potential should not be excluded routinely from participation in clinical research.

Provide the following information:

- A description of the subject selection criteria and rationale for selection in terms of the scientific objectives and proposed study design;
- A compelling rationale for proposed exclusion of any sex/gender or racial/ethnic group;
- The proposed dates of enrollment (beginning and end);
- The proposed sample composition of subjects.

You may reference grant application/sponsor's relevant protocol pages and attach as an appendix using the below attachment button, however, a summary paragraph must be provided in the text box below.

This study will aim to enroll 60 participants

Inclusion Criteria:

• Male or female between the ages of 18 and 40
• Participants must be overweight to obese (BMI 25–35 kg/m2) Not pregnant or lactating or planning to become pregnant in the next 6 months
• No limitations to safely participating in physical activity

Exclusion Criteria:
• Currently dieting to lose weight and no weight loss or gain >2kg over the past 3 months,
• Taking any medications that affect energy expenditure or eating,
• Regularly exercising (planned and structured aerobic or resistance activity).
• Participants must have no major health problems, cannot have known cardiovascular (cardiac, peripheral vascular, cerebrovascular), pulmonary (COPD, interstitial lung disease, cystic fibrosis) or metabolic (diabetes, thyroid disorders, renal or liver disease) disease.
• Tobacco use
• Have a medical condition that prevents safe exercise.

Appropriateness of exercise will be screened using a Health History and Physical Activity Readiness Questionnaire. Subjects will taste and rate their liking of the study foods. Subjects must have a liking of at least 5 out of 10 for 50% of the study foods. Subjects will provide informed consent before participation. There are no hypotheses on gender or race effects and we will strive to recruit a representive sample that includes all gender and ethnic groups.

Attachments

Subject Recruitment Methods & Privacy: Using active voice, describe plans for the identification and recruitment of subjects, including how the population will be identified, and how initial contact will be made with potential subjects by those having legitimate access to the subjects' identity and the subjects' information.

Describe the setting in which an individual will be interacting with an investigator or how and where members of the research team will meet potential participants. If applicable, describe proposed outreach programs for recruiting women, minorities, or disparate populations as participants in clinical research. Describe steps taken to minimize undue influence in recruiting potential participants.

Please note: Based upon both legal and ethical concerns, the UK IRB does not approve finder's fees or "cold call" procedures made by research staff unknown to the potential participant.

For additional details, see topic "Recruitment of Subjects/Advertising" on ORI's IRB Survival Handbook web page and the PI Guide to Identification and Recruitment of Human Subjects for Research [PDF].

Various media will be used to advertise the study and recruit subjects from the greater Lexington metropolitan area and surrounding communities. This may include brochures, flyers (APPENDIX 1), and print media such as the Lexington Herald-Leader. Internet advertisements including Facebook ads on UK_CCTS, Research Depts, UK HealthCare, and Research Match (APPENDIX 2 a/b/c) will be utilized. Potential subjects will complete an online screening application (RedCap, APPENDIX 3). Dr. Flack or his designee will contact each potential subject to inform them if they are deemed initially eligible based on study entry criteria and invite them to an information/screening meeting.

Eligible applicants will be invited to an information/screening appointment at Dr. Flack's lab space in the Funkhouser Building (room 117).

Back to Top

Advertising: Specify if any advertising will be performed. If yes, please see "IRB Application Instructions - Advertisements" for instructions on attaching copies of the information to be used in flyers or advertisements. Advertisements must be reviewed and approved by the IRB prior to use. For additional details, see topic "Recruitment of Subjects/Advertising" on ORI's IRB Survival Handbook web page for the *PI Guide to Identification and Recruitment of Human Subjects for Research* [D7.0000] document [PDF]. If you will be recruiting subjects via advertising at non-UK owned or operated sites, you should include a copy of written permission from that site to place the advertisement in their facilities.

Note: Print and media advertisements that will be presented to the public also require review by UK Public Relations (PR) to ensure compliance with UK graphic standards, and equal opportunity language.

Advertisements attached

Attachments

Attach Type	File Name
Advertising	AGDHN-008_monitor(PR).pdf
Advertising	AGDHN-008_flyer(PR).pdf
Advertising	AGDHN-008_RM(PR).pdf

45819
CLOSED

Informed Consent Process: Using active voice, describe the consent/assent procedures to be followed, the cir[illegible] which consent will be sought and obtained, the timing of obtaining informed consent, whether there is any waiting period between informing the prospective subject and obtaining consent, who will seek consent., steps taken to minimize the possibility of coercion or undue influence, the method used for documenting consent, and if applicable who is authorized to provide permission or consent on behalf of the subject. Note: all individuals authorized to obtain informed consent should be designated as such in the E-IRB "Study Personnel" section of this application.

Describe provisions for obtaining consent/assent among any relevant special populations such as children (see Children in Research Policy [PDF] for guidance), prisoners (see Summary of Prisoner Regulations [PDF] for guidance), and persons with impaired decisional capacity (see Impaired Consent Capacity Policy [PDF] for guidance). Describe, if applicable, use of specific instruments or techniques to assess and confirm potential subjects' understanding of the nature of the elements of informed consent and/or a description of other written materials that will be provided to participants or legally authorized representatives. If you have a script, please prepare it using the informed consent template as a guide, and submit it on a separate page.

Informed Consent for Research Involving Emancipated Individuals
If you plan to enroll some or all prospective subjects as emancipated, consult with UK legal counsel **when preparing the IRB application and prior to submitting the application to the IRB**. Include legal counsel's recommendations (legal counsel's recommendations may be attached in the E-IRB "Additional Information" section as a separate document, if necessary). For a complete definition of emancipated minors, see the section on *Emancipated Individuals* in the Informed Consent SOP [PDF].

Informed Consent for Research Involving Non-English Speaking Subjects
If you are recruiting non-English speaking subjects, the method by which consent is obtained should be in language in which the subject is proficient. Describe the process for obtaining informed consent from prospective subjects in their respective language (or the legally authorized representative's respective language). In order to ensure that individuals are appropriately informed about the study when English is their second-language, describe a plan for evaluating the level of English comprehension, and the threshold for providing a translation, or explain why an evaluation would not be necessary. For additional information on inclusion of non-English speaking subjects, or subjects from a foreign culture, see IRB Application Instructions for Recruiting Non-English Speaking Participants or Participants from a Foreign Culture.

Research Repositories
If the purpose of this submission is to establish a research repository describe the informed consent process. For guidance regarding consent issues, process approaches, and sample language see the Sample Repository/Registry/Bank Consent Template [PDF]

6. Informed Consent Process: Prior to entering the study, the risks and benefits of participating will be explained to participants by a member of the study team authorized to obtain informed consent. Written informed consent forms will be placed in a locked file cabinet in a separate room from other study files. The study participant will receive a copy of the informed consent form.

Back to Top

Research Procedures: Describe the research procedures that will be followed. Identify all procedures that will be carried out with each group of subjects. Differentiate between procedures that involve standard/routine clinical care and those that will be performed specifically for this research project.

Please see Appendix 4 for the study Schedule of Events Table. All test and procedures listed are being done for the purpose of the research study.

During the initial visit, participants will be informed about the study and have any questions answered. If deemed eligible and the participant desires to participate, they will be asked to provide written informed consent. After signing the consent, participants will fill out a demographic form (APPENDIX 5), a medical history form and Physical Activity Readiness form (APPENDIX 6), and be measured for anthropometrics (height and weight). These forms will be stored in a locked file cabinet in Dr. Flack's office separate from other study files. Subjects will also be provided an ActiGraph device to wear for the following week to monitor free-living physical activity and complete the Preference for and Tolerance of the Intensity of Exercise Questionnaire (PRETIE-Q) (APPENDIX 7) [25, 26], the Behavioral Regulation in Exercise Questionnaire (BREVQ-3) (APPENDIX 8) [27], and the vendor form (APPENDIX 9), so a payment can be processed) at this time.

Outcome visits: Outcome measures will be completed during baseline and immediately following the 12 weeks of training (Post/ week 13). Outcome measures will be completed at the CCTS, laboratory space in Funkhouser (room 117), Joe Craft Football Training Facility, or the Human Performance Laboratory as needed. The sequential order of the visits to measure outcomes is flexible in order to meet the scheduling complexities of participants.

Accelerometer administration: In Funkhouser 117, occurs at screening, after post-training assessments (week 14), and after follow-up assessments (week 19). Subjects will wear an accelerometer for seven consecutive days after receiving the accelerometer to measure usual physical activity. This will be scheduled so no exercise tests or sessions will fall on the 7 days wearing the monitor. This will be administered on participants' initial visit to Funkhouser 117 where they will also sign the consent form.

Surveys, taste test, height, weight: Will occur at screening, post-training/week 13, and follow up/week 18. The taste test will be used to determine the foods to be used in the RRVfood test. This visit will occur in Funkhouser 117.

RRVfood test: Will occur at weeks -1 or -2, 13, and 18. Subjects will report 2 to 3 hours post-prandial to room 117 Funkhouser and complete a task where they will earn points towards their chosen foods.

45819
CLOSED

RRVexercise test: Will occur at weeks -2 or -3, 13, and 18 at the Human Performance Laboratory (MDS, B04). Subjects will sample for 2 minutes each and rate their liking of the 3 exercise options (treadmill, elliptical ergometer, or cycle ergometer) and sedentary alternatives (magazines, puzzles, card games). This testing can take place in the morning or afternoon, but will occur at the same time of day for a given subject.

BodPod + RMR test: Will occur at weeks -1 or -2, and 13. Subjects will report at least 10 hours post-prandial and be assessed for their body composition (BodPod) and resting metabolic rate (RMR) at Funkhouser 117.

CCTS Meal test visit Will occur at weeks -1 or -2, and 13. Subjects will arrive to the CCTS at least 10 hours post-prandial and have an IV-catheter placed and fasted blood sample collected. Subjects will then be administered a standardized breakfast and have blood drawn immediately after breakfast (-2.75), and every 15 minutes during the first hour (-2.5, -2.25, -2 hr) and then every 30 minutes thereafter (-1.5, -1, -0.5, 0) for a total testing time of 3 hrs. Subjects will also complete VAS scales for hunger and satiety at each blood draw time point (APPENDIX 10).

CCTS Exercise test and DXA visit Will occur at weeks -1 or -2, week 6, week 13
Participants will report to the CCTS to perform a graded exercise test to determine kcal expended per minute at different heart rate intensities using indirect calorimetry. This information will be used to set the intensity and duration of their exercise training sessions during the intervention period of the study. This will be performed at baseline, on weeks 6 - 7, and after training. The mid-point measure will be used to adjust the duration and intensity of the exercise training sessions to account for any improvements in aerobic fitness. The post-time point measure will be used to determine aerobic fitness improvements. Prior to this test participants will also have a DXA scan preformed.

Additional DEXA test: Will occur at weeks -1 or -2 and 13. Subjects will report to the Joe Craft Football Training Facility to have an additional DXA performed on the UK athletics' Norland model DXA, a different model than the "gold standard" iDXA used at the CCTS.

Food Diary: for weeks -3, 4, 8, 12, 17 participants will record all foods they eat with details on quantity and cooking methods in a dietary intake record (APPENDIX 11).

Exercise session download meeting: Once per week during the exercise intervention (weeks 1-12) participants will return to Funkhouser 117 to have their exercise sessions for the previous week downloaded to ensure compliance.

Intervention: For the exercise treatments, participants will engage in aerobic exercise (treadmill, stationary bike, elliptical) to induce a 3000 kcal/week energy expenditure exercising either 6 sessions per week (500 kcal per session) or 2 sessions per week (1500 kcal per session). The exercise intervention will last 12 weeks, followed by one week of post-testing and an unsupervised 4-week period where participants may exercise or not before final follow-up assessments at week 17. Exercise will occur at a moderate to difficult intensity and prescribed based on individual rates of energy expenditure (kcal/min) at different heart rate zones. Most exercise will occur at 65% of HRR, an intensity commonly prescribed for sedentary individuals beginning an exercise program [28]. The 6 session per week group should be able to complete the session in 20 to 60 min, while the 2 session per week group may take between 1.5 and 2.5 hours based on how many calories subjects burn exercising at 65% HHR. Subjects can take rest breaks as necessary during the exercise sessions. The first 2 to 5 exercise sessions will be supervised by research staff to introduce subjects to the use of the equipment and to ensure participants will be able to exercise for the prescribed time to meet their energy-expenditure goal. Then, exercise sessions will be supervised on an as needed basis in order to encourage compliance. To verify treatment implementation, subjects will wear a chest-strap heart rate monitor. Missed sessions (unless due to injury or illness) will be compensated for during the following sessions or made-up on another day. Heart rate and workout data will be reviewed by the research staff every week. Subjects will be able to use a 12-week pass to the Lexington-area YMCA(s) to assure access to exercise facilities if needed.

Measurements

Height and Weight. Body weight will be measured with a Tanita bioelectrical impedance scale after voiding. Subjects will wear scrubs or shorts and a t-shirt and no shoes when being weighed. Height will be with a stadiometer and standard anthropometric technique. These measurements will occur at screening to determine if subjects meets BMI inclusion criteria.

Food reinforcement task. Subjects will report 2-4 hr post-prandial to Funkhouser 117. RRVfood is assessed by evaluating the number of responses (mouse button presses) a participant is willing to complete to gain access to food. The foods used in the task (Table 1) were chosen to allow subjects to choose foods that are often considered both "junk" foods and "healthy" snacks with similar nutrient densities. The experimental environment will include two personal computers on separate workstations. At one station is the computer on which the participant can earn points towards their most liked junk food, with the other station being set up for them to earn points for their most liked healthy food. The most liked foods of each category are determined at screening by tasting a small portion of each food and rating each using a Likert-type scale (APPENDIX 12). Participants can freely go back and forth between stations. The program used for the reinforcement task presents a game that mimics a slot machine. A point is earned each time the 3 shapes that appear with each mouse button press match in shape and color. Access to the alternatives is dependent on earning points, which can be exchanged for access to the reinforcers. For every 5 points earned, the subject will receive a 70-100 kcal portion of the food they were working for. Points are delivered on independent progressive fixed-ratio schedules. The schedules of reinforcement for food and reading begin at a fixed ratio of 4 and then doubles (8, 16, 32, […] 2048) each time 5 points are earned. For instance, the participant initially has to click the mouse four times to earn each point, but after the first portion is earned, then 8 clicks are required to earn each of the 5 points, and so on. The reinforcer not earned does not increase to the next level until it is earned. The testing session will end when participants no longer wish to earn points for access to either food choice. Water will be provided ad libitum. Participants are instructed on how to complete the computer task and will complete a practice session to

45819
CLOSED

demonstrate their understanding of the task. The task is completed with the experimenter in an adjacent room. Ou [illegible] total number of responses (mouse-presses) performed for each type of food and the Pmax [29] of each choice, which is the last schedule that a participant met the response requirements for access to each food choice.

Table 2. Foods to be used in the RRV of food task

	E density	Protein	Carbohydrate	Sugar	Fat
	kcal/g	% of E	% of E	% of E	% of E
Doretos chips	5.4	5.2	48.0	2.7	48.0
Oreo cookies	4.7	4.7	60.2	35.0	38.0
Snickes bar	4.9	6.2	50.2	41.2	43.6
Nutri-grain bar	3.2	6.1	73.3	80.0	20.6
Banana chips	5.2	0.02	42.8	6.8	55.7
Mixed nuts	6.1	12.3	12.5	0.03	75.2

Physical activity reinforcement task. To assess the reinforcing value to be physically active, one computer station is set-up for the subject to earn access to physical activity (treadmill, elliptical ergometer, cycle ergometer) and the other computer station is set to earn access to sedentary alternatives (magazines, puzzles, card games). Access to the alternatives is dependent on earning points, which can be exchanged for the reinforcers using the schedules of reinforcement described above. For every 5 points earned, the subject will receive 5 min of access to exercise or 5 min of time to engage in the sedentary behavior, depending on the reinforcer that they were working for. Prior to the test participants will rate how much they like the exercise options (APPENDIX 13) and the sedentary alternative (APPENDIX 14) This sedentary alternative is provided to reduce the likelihood that participants would engage in responding for exercise out of boredom. This test will take place in the Human Performance Laboratory.

Body Composition. Body composition will be measured using a GE Lunar iDXA machine at the CCTS. A total body scan will be conducted with participants lying supine on the table and arms positioned to the side and palms flat on the table. Scans will be completed using the standard mode, unless the thick scan mode is suggested by the software. All scans will be analyzed using GE Lunar enCORE Software (13.60.033). Automatic edge detection will be used for scan analyses. The machine will be calibrated before each scanning session, using the GE Lunar calibration phantom. Outcomes will include total body lean mass, fat mass, bone mineral content and bone mineral density.
Each subject will undergo total body composition testing using a total body dual-energy x-ray absorptiometry (DXA) scan, and have their regional fat estimations performed using 3 circumference measures. Total body and regional, absolute and relative measures of soft tissue (fat, fat-free and mineral-free lean) masses will be made and analyzed by DXA scanning using a Lunar iDXA (GE Lunar Inc., Madison, WI; software version 13.10) using standardized procedures. The Lunar iDXA is the machine that is used during standard clinical testing. The waist, abdominal, and hip circumference measurements will be performed in triplicate using a flexible steel anthropometric tape (Rosscraft Anthrotape; Rosscraft Innovations Inc.) using standardized procedures. The body composition assessments testing will take place in the University of Kentucky Clinical Services Core (CSC) Functional Assessment and Body Composition Core (FAABC; inpatient suit 5th floor Chandler Medical Center) Laboratory of the Center for Clinical and Translational Science (CCTS).

Additional body composition tests will be performed on the Department of Dietetics and Human Nutrition’s BodPod (air-displacement plethysmography) and the UK Athletics’ DXA (Norland model) as a separate measure of internal reliability of these machines by comparing to the iDXA. The iDXA is the “gold standard” and will be used as the primary measure to test the current hypotheses.

Aerobic Fitness. A graded exercise treadmill test will be used to test aerobic fitness in addition to calculating rate of energy expenditure. Oxygen consumed and expired CO_2 will be analyzed by indirect calorimetry. The test begins with a 5 minute warm-up of walking at 0% grade, 3.0 mph. Upon completion of the warm-up, the grade increases to 2.5% for 3 minutes with the speed fixed at 3.0 mph. The treadmill grade will then increase every 3 minutes to produce an approximately 10 beat/minute increase in heart rate from the previous stage. The test will continue until a heart rate of 85% HRR was attained or the participant fells they can no longer continue. The treadmill test will be performed at baseline, during week 6, and post training. The week 6 measurements will be used to adjust the intensity and duration of the exercise training sessions to account for improvements in aerobic fitness. Performance work capacity (PWC) will be utilized as a measurement of sub-maximal aerobic fitness with the rates of oxygen consumed at heart rates of 150 and 170 beats/minute serving as aerobic fitness outcomes.

Resting Metabolic Rate. Subjects will arrive to Funkhouser 117 fasted one morning prior to beginning the intervention (baseline) and 48 or more hours after their final exercise session of the intervention (week 13) to determine their RMR via indirect calorimetry (Cosmed USA, Chicago). This test lasts 45 minutes. Resting heart rate will be determined during the RMR test to be used to calculate HHR.

Hormone measurement. Subjects will report fasted to the CCTS and have an IV catheter placed to provide a fasted blood sample to analyze hormone and peptide concentrations associated with hunger and body weight regulation and complete VAS to determine hunger and satiety immediately prior to a standardized breakfast. The standardized breakfast will provide 20% of estimated energy needs for each individual based on their RMR and a sedentary activity factor of 1.4. Breakfast will consist of 2% milk and cornflakes (with the option of substituting unsweetened soy milk for those with a lactose sensitivity). Breakfasts will be consumed within 15 minutes. VAS scales for hunger and satiety and blood draws for hunger hormone measurement will be completed immediately before breakfast (-3hr) immediately after (-2.75), and every 15 minutes during the first hour (-2.5, -2.25, -2 hr) and then every 30 minutes thereafter (-1.5, -1, -0.5, 0) [30, 31]. Blood samples will be collected in EDTA-coated and serum tubes. Serum GLP-1, leptin, glucagon, and active grehlin will be measured using ELISA (Millipore, Phoenix Pharmaceuticals, Alpco). Serum insulin will be

measured with a chemiluminescent immunometric assay (Siemens). Samples will be batch-analyzed to reduce in[illegible] and stored at the CCTS until the conclusion of the study.

Habitual physical activity. For a 7 day period starting the day after subject's screening, 7 days following post-testing outcome assessments, and for 7 days during week 17, an ActiGraph accelerometer (GT3XPB model; Pensacola, Florida) will be worn to provide information regarding changes in habitual, free-living physical activity. Subjects will be instructed to wear it at the hip using the provided belt during all hours awake except when bathing or swimming. Data will be cleaned of non-wear time, defined as consecutive strings of zeros greater than 20 minutes. An epoch of 10 seconds will used for data collection. This will be used to measure weekly minutes of moderate to vigorous physical activity (MVPA) as well as minutes of sedentary activity and light intensity activity using the Crouter et.al algorithm [32], and Freedson cut-points [33].

Treatment Adherence. Subjects will be instructed to follow specific exercise plans that will elicit a 3,000 kcal energy expenditure per week. The length of the exercise session will depend on each individual's rate of energy expenditure. Each subject will be given a Polar heart rate monitor device to wear for each training session and meet with a research staff member weekly to download the previous week's exercise sessions to monitor compliance.

Tolerance for the discomfort of exercise. Discomfort tolerance will be measured by questionnaires. Subjects will complete the validated Preference for and Tolerance of the Intensity of Exercise Questionnaire (PRETIE-Q, [34, 35] (APPENDIX 7), at baseline and after training.

Energy and macronutrient intake. During weeks -3, 4, 8, 12, 17, participants will record their dietary intake for seven consecutive days.

Calculations. The accumulated energy balance (AEB) will be calculated from changes in FM and FFM as body composition changes reflect long-term alterations in energy balance (Rosenkilde et al. 13). Gains of 1kg FM or 1kg FFM will reflect 12,000 and 1,780 kcal, respectively (Elia et al., 2003). Losses of 1kg FM or 1kg FFM will be assumed to equal 9,417 and 884 kcal, respectively (Forbes, 1990).
Exercise energy expenditure (ExEE) is calculated from the training-induced energy expenditure (TrEE) of 300 or 600 kcal/session with the addition of 15% excess post-exercise energy expenditure (Bahr, et al., 1997). The resting energy expenditure (REE) that would have occurred during the exercise sessions (REE x 1.2) will be subtracted. Thus, ExEE = (TrEE x 0.15) + (TrEE – training duration x REE 1.2) (Rosenkilde et al. 13).

The degree of compensation in response to the increase in ExEE is assessed through a compensation index calculated as (ExEE – AEB)/ExEE x 100%. When the compensation index equals zero AEB equals ExEE, or changes in body composition equal calories expended during exercise. Positive compensation suggests changes in body composition indicating a less negative energy balance than expected, whereas a larger than expected negative energy balance is referred to as negative compensation. Degree of compensation = (ExEE – AEB)/ExEE x 100%.

Statistical Analysis Plan: Baseline demographic and physical characteristics will be reported as means and standard errors. Baseline group (6 days/week, 2 days per week exercise, sedentary control) differences will be tested with one-way ANOVA. Alterations in accumulated negative energy balance, body composition, RRVfood, RRVexercise, dietary intake, energy expenditure, exercise discomfort, hunger/satiety hormones, and fitness will be tested with two-way repeated measures analysis of variance (RMANOVA) with group treated as a between-subjects variable and time treated as a within subjects variable. Power Analysis: The primary outcome is accumulated negative energy balance. Based on previous research the mean accumulated negative energy balances for 300 kcal of exercise and 600 kcal of exercise conditions were -83% + 100% (mean + SD) and 20% + 50%, respectively.

Attachments

Data Collection: List the data or attach a list of the data to be collected about or from each subject (e.g. interview script, survey tool, data collection form for existing data).

If the research includes survey or interview procedures, the questionnaire, interview questions or assessment scales should be included in the application (use attachment button below).

The data collection instrument(s) can be submitted with your application in draft form with the understanding that the final copy will be submitted to the IRB for approval prior to use (submit final version to the IRB for review as a modification request if initial IRB approval was issued while the data collection instrument was in draft form).

Note: The IRB approval process does not include a statistical review. Investigators are strongly encouraged to develop data management and analysis plans in consult with a statistician.

attached are the tools used to collect data from each subject.

Attachments

Attach Type	File Name
DataCollection	App3_Screening Application.docx
DataCollection	App5_DemographicQ.docx
DataCollection	App6_MedhistPARQ.docx
DataCollection	App7_PRETTIE-Q.docx
DataCollection	App9_vendorform.pdf

DataCollection	App8_BREVQ3.docx
DataCollection	App10_VAS scales.docx
DataCollection	App11_Food intake record.pdf
DataCollection	App12_Liking of foods.docx
DataCollection	App13_Liking of Exercise.docx
DataCollection	App14_Liking of Sedentary alt.docx
DataCollection	EXERCISE REGULATIONS QUESTIONNAIRE.docx

Resources: Describe what resources/facilities are available to perform the research (i.e., staff, space, equipment). Such resources may include a) staffing and personnel, in terms of availability, number, expertise, and experience; b) psychological, social, or medical services, including counseling or social support services that may be required because of research participation; c) psychological, social, or medical monitoring, ancillary care, equipment needed to protect subjects; d) resources for subject communication, such as language translation services, and e) computer or other technological resources, mobile or otherwise, required or created during the conduct of the research. Please note: Some mobile apps may be considered mobile medical devices under FDA regulations (see FDA Guidance). Proximity or availability of other resources should also be taken into consideration, for example, the proximity of an emergency facility for care of subject injury, or availability of psychological support after participation.

Research activities conducted at performance sites that are not owned or operated by the University of Kentucky, at sites that are geographically separate from UK, or at sites that do not fall under the UK IRB's authority, are subject to special procedures for coordination of research review. Additional information is required (see IRB Application Instructions - Off-Site Research web page); supportive documentation can be attached in the E-IRB "Additional Information" section. Provide a written description of the role of the non-UK site(s) or non-UK personnel who will be participating in your research. The other site may need to complete its own IRB review, or a cooperative review arrangement may need to be established. Contact the Office of Research Integrity at (859) 257-9428 if you have questions about the participation of non-UK sites/personnel.

If the University of Kentucky is the lead site in a multi-site study, or the UK investigator is the lead investigator, describe the plan for managing the reporting of unanticipated problems, noncompliance and submission of protocol modifications and interim results from the non-UK sites.

The proposed study will utilize CCTS staff and facilities for measurement of hunger/satiety hormone concentrations, body composition (DEXA), and aerobic fitness tests. Laboratory space at Funkhouser includes a Quark RMR measurement equipment (Cosmed), NDS-R software for nutrition analysis, BodPod for air displacement plethysmography, and computers and space necessary for the food reinforcement task. The reinforcing value of exercise measurement will take place in the Human Performance Laboratory, where treadmills and cycle ergometers are available for participant use. The Department of Dietetics and Human Nutrition recently purchased new Polar A-300 heart rate monitors and ActiGraph accelerometers for the proposed study. Dr. Flack (PI) has led similar studies and may be training up to three graduate students that will assist in conducting the study.

Potential Risks: Describe any potential risks or likely adverse effects of the drugs, biologics, devices or procedures subjects may encounter while in the study. Please describe any physical, psychological, social, legal or other risks and assess their likelihood and seriousness.

DXA Scan. All subjects will be informed of the small amount of radiation they will receive while going through this procedure. Female subjects of childbearing potential will receive a urine pregnancy test prior to undergoing the DXA Scan. Those women found to be pregnant will not undergo the scan and will be removed from the study.

Blood Draw/ placement of IV catheter: Upon placement of the IV catheter, there may be some slight discomfort experienced from the insertion of the needle into the vein. Potential infection, soreness, pain, bleeding, bruising, and fainting may occur. This pain and soreness may last up to 24 hrs following the procedure. A trained nurse will place the IV catheter and standard precautions will be taken to guard against the following potential risks.

Possible Risk/Side Effect How often has it occurred? How serious is it? Can it be corrected?
Soreness It occasionally occurs Can be easily treated Yes
Pain It occasionally occurs Does not impact your overall health It will go away within 24 hrs
Bleeding It occasionally occurs Can be easily treated Yes, by applying pressure
Bruising It occasionally occurs Can be easily treated Yes
Fainting It is uncommon Can be easily treated by lying down with the legs elevated Yes, usually in 20 minutes
Infection It is very uncommon Can be treated Yes

Back to Top

Safety Precautions: Describe the procedures for protecting against or minimizing any potential risks, *including risks of breach of confidentiality or invasion of privacy*. Where appropriate, discuss provisions for ensuring necessary medical or professional intervention in the event of adverse events, or unanticipated problems involving subjects. Also, where appropriate, describe the provisions for monitoring the data collected to ensure the safety of subjects. If vulnerable populations other than adults with impaired consent capacity are to be recruited, describe additional safeguards for protecting the subjects' rights and welfare.

Provisions to guard against the potential risks and discomforts discussed in section 9 are as follows: Every precaution to prevent a direct study injury will be taken by medical personnel and the investigators. Should an adverse or serious adverse event occur, the research participant will be followed by physicians, registered nurses and other research staff members for the duration of the

45819
CLOSED

participant's hospitalization. Routine care will be provided by the hospital staff. Emergency medical equipment, m[illegible] supplies will be at the physician's disposal should the participants have an acute untoward reaction.

Benefit vs. Risk: Describe potential benefits to the subject(s); include potential benefits to society and/or general knowledge to be gained. Describe why the risks to subjects are reasonable in relation to the anticipated benefit(s) to subjects and in relation to the importance of the knowledge that may reasonably be expected to result. If you are using vulnerable subjects (e.g., impaired consent capacity, pregnant women, etc…), justify their inclusion by describing the potential benefits of the research in comparison to the subjects' vulnerability and the risks to them. For information about inclusion of certain vulnerable populations, see the IRB/ORI Standard Operating Procedure for Protection of Vulnerable Subjects [C3.0100] [PDF].

Participants enrolled in the present study will gain insight into factors that influence weight loss with exercise and are expected to experience a decrease in body weight and increase in physical fitness. Risks associated with exercise (injuries, cardiac arrest) will be minimized by careful prescription of exercise and weekly monitoring by Dr. Flack and his staff. Other risks are as follows:

• Questionnaires: You may get frustrated when doing the surveys. Some questions may be sensitive, and you may become upset. If you become upset by questions you may stop at any time or choose not to answer a question.
• Exercise and Fitness Tests: You may not like participating in the exercise that is required. There is a small risk of sprains, strains, and broken bones as the result of exercise. To reduce this risk, you will be supervised by experienced staff. Some soreness may occur 24-48 hours after exercise but this will go away with time. Exercise can uncover or worsen hidden heart problems such as not enough blood flow to the heart muscle and irregular beats. It is unlikely you will have problems with your heart or circulatory system. Should you develop symptoms of any medical problems, testing will be stopped immediately.
• Armbands and chest straps: There is a chance of skin irritation from the armband and the chest strap. These devices will be cleaned between uses, and trained staff will instruct you on how to properly wear the devices. If any discomfort occurs, please report it to the research staff immediately and you will be instructed to remove the device or adjust its position.

Available Alternative Treatment(s): Describe alternative treatments and procedures that might be advantageous to the subjects, should they choose not to participate in the study. This should include a discussion of the current standard of care treatment(s).

There are no available alternative treatments if participants wish not to participate.

Back to Top

Research Materials, Records and Privacy: Identify the sources of research material obtained from individually identifiable living human subjects. Indicate what information (specimens, records, data, genetic information, etc.) will be recorded and whether use will be made of existing specimens, records or data. Explain why this information is needed to conduct the study.

Return of Research Results or Incidental Findings (if applicable):
If research has the potential to identify individual results or discover incidental findings that could affect the health of a subject, describe plans to assess, manage, and if applicable disclose findings with individual subjects or provide justification for not disclosing. For IRB expectations, refer to the UK IRB "Frequently Asked Questions (FAQs) on the Return of Research Results or Incidental Research Findings" [PDF].

The investigative team maintains the right to keep, preserve, use and dispose of the findings of this investigation in accordance with University of Kentucky IRB and Records Management guidelines. Officials of the University of Kentucky maintain the right to inspect the records of the study at any time. Investigational records from this study will be maintained in a confidential manner; participant names will not be associated with any published results.

Human subjects will provide a blood sample to be stored at the CCTS facilities. These samples will be assessed for concentrations of certain hormones and reported to Dr. Flack. These samples will not be used for another study.

Confidentiality: Specify where the data/specimens will be stored and how the researcher will protect both the data and/or specimens with respect to privacy and confidentiality. Address physical security measures (e.g., locked facility, limited access); data security (e.g., password-protection, data encryption); safeguards to protect identifiable research information (e.g., coding, links, certificate of confidentiality); and procedures employed when sharing material or data, (e.g., honest broker (if applicable), written agreement with recipient not to re-identify). If you plan to procure, store, and/or share material (tissue/specimens/data) expressly for use in current or future research, describe measures that you will take to secure and safeguard confidentiality and privacy.

Describe whether data/specimens will be maintained indefinitely or destroyed. If maintained, specify whether identifiers will be removed from the maintained information/material. If identifiers will not be removed, provide justification for retaining them. If the data/specimens will be destroyed, describe how and when the data/specimens will be destroyed [Note: The investigator is responsible for retaining the signed consent and assent documents and IRB research records for at least six years after study closure as outlined in the Study Closure SOP [PDF]. If the research falls under the authority of FDA or other regulatory agency, the investigator is responsible for retaining the signed documents and IRB records for the period specified if longer than six years after completion of the study]. For multi-site studies, the PI consults the study sponsor regarding retention requirements, but must maintain records for a minimum of six years after study closure. Also, specify who will access the identified data/specimens, and why they need access. If applicable, describe what measures will be taken to ensure that subject identifiers are not given to the investigator. If applicable, describe procedures for sharing data/specimens with entities not affiliated with UK.

NIH-funded genomic research: The National Institutes of Health (NIH) Genomic Data Sharing (GDS) Policy sets forth

45819
CLOSED

expectations that ensure the broad and responsible sharing of genomic research data consistent with the inf[illegible] of study participants from which the data was obtained. If you are submitting genomic data to an NIH data repository, describe your NIH data sharing plan.

Please note: The IRB expects researchers to access the minimal amount of identifiers to conduct the study and comply with applicable HIPAA and Family Educational Rights and Privacy Act (FERPA) requirements. If data are going to be collected, transmitted, and/or stored electronically, for appropriate procedures please refer to the guidance document "Confidentiality and Data Security Guidelines for Electronic Data" [PDF].

Also please note that storage of data on cloud services may not be appropriate and is subject to applicable university policies regarding the use of cloud services. If deemed too sensitive or inappropriate to be stored or collected using cloud services, the IRB may require an alternate method of data storage in accordance with applicable university policies and the electronic data security guidance document referenced above.

If a research protocol involves the creation and/or use of a computer program or application, mobile or otherwise, please specify whether the program/application is being developed by a commercial software developer or the research team and provide any relevant information regarding the security and encryption standards used, how data is stored and/or transmitted to the research team, what information about the subjects the program/application will collect, etc. For relevant information to include, see Considerations for Protocol Design Concerning Digital Data [PDF]. The IRB may require software programs created or used for research purposes be examined by a consultant with appropriate Internet technology expertise to ensure subject privacy and data are appropriately protected.

Back to Top

Human subjects will provide a blood sample to be stored at the CCTS facilities. These samples will be assessed for concentrations of certain hormones and reported to Dr. Flack. These samples will not be used for another study.

All specimens, forms, and electronic files (exercise sessions, RRV reports) will be coded with a unique, unidentifiable code for each participant. Blood specimens will be stored in a secured, locked freezer at the CCTS, locked file cabinets will be utilized to store subject files, and a password protected computer will be used to protect electronic files. Only personal listed will access study data for statistical analysis, record keeping, and monitoring. Blood specimens will be immediately destroyed after determination of hormone concentrations according to appropriate bio-hazard disposal procedures. Other study files will be kept for 6 years after completion of the study.

Information will be combined with information from other people taking part in the study. When we write about the study to share it with other researchers, we will write about the combined information we have gathered. Participants will not be personally identified in these written materials. We may publish the results of this study; however, we will keep their name and other identifying information private.

There are some circumstances in which we may have to show their information to other people. For example, the law may require us to show their information to a court or to tell authorities if they report information about a child being abused or if they pose a danger to themselves or someone else.

Study, medical charts and any other items containing confidential items will be stored in a safe place overnight and not left on desks. Charts will not be left in an area where others might have access to them.

In order to process study payments, we will need collect their social security number. The do not have to give us this number however, refusing to provide their social security number may result in their not receiving study payment.

Participant confidentiality is strictly held in trust by the participating investigators, their staff, and the sponsor(s) and their agents. This confidentiality is extended to cover testing of biological samples in addition to the clinical information relating to participants. Therefore, the study protocol, documentation, data, and all other information generated will be held in strict confidence. No information concerning the study or the data will be released to any unauthorized third party without prior written approval of the IRB and principal investigator.

Authorized representatives of the IRB and the CCTS may inspect all documents and records required to be maintained by the investigator, including but not limited to, medical records (office, clinic, or hospital) for the participants in this study. The clinical study site will permit access to such records.

The study participant's contact information will be securely stored at each clinical site for internal use during the study. At the end of the study, all records will continue to be kept in a secure location for as long a period as dictated by the IRB and Institutional regulations.

Study participant research data, which is for purposes of statistical analysis and scientific reporting, will stored with the principal investigator. This will not include the participant's contact or identifying information. Rather, individual participants and their research data will be identified by a unique study identification number. We will collect all data on an Excel/RedCap Dataset

The study information collected from the participation in the study will be entered into a secure server (REDCap).

Officials of the University of Kentucky, The Center for Clinical and Translational Science, may look at or copy pertinent portions of records that identify the participant.

Back to Top

Payment: Describe the incentives (e.g., inducements) being offered to subjects for their time during participation in the research study. If monetary compensation is offered, indicate how much the subjects will be paid and describe the terms and schedule of payment. (It is IRB policy that provision should be made for providing partial payment to subjects who withdraw before the completion of the research. Monetary payments should be prorated or paid in full.)

Subjects will be payed $150 for completion of the study to be paid all at once at the conclusion of the study. Should a participant withdraw from the study before completing the intervention or post-testing assessments they will compensated $50 upon withdrawal. If participants do not complete baseline measurements before withdrawing they will not be compensated. Participants can expect to receive a check in the mail approximately 4-6 weeks following the study visit.

Costs to Subjects: Describe any costs for care associated with research (including a breakdown of standard of care procedures versus research procedures), costs of test drugs or devices, and research procedure costs that are the subject's responsibility as a consequence of participating in the research. Describe any offer for reimbursement of costs by the sponsor for research related injury care.

There is no cost to the participant for taking part in this study. The cost associating with care and treatment for any research related injury will the responsibility of the participant or their insurance company.

Data and Safety Monitoring: The IRB requires review and approval of data and safety monitoring plans for greater than minimal risk research, clinical research, or NIH-funded/FDA-regulated clinical investigations.

If you are conducting greater than minimal risk research, clinical research, or your clinical investigation is NIH-funded/FDA-regulated, describe your Data and Safety Monitoring Plan (DSMP). Click here for additional guidance on developing a Data and Safety Monitoring Plan.

If this is a *non-sponsored investigator-initiated* protocol considered greater than minimal risk research, clinical research, or your clinical investigation is FDA-regulated, *and* if you are planning on using a Data and Safety Monitoring Board (DSMB) as part of your DSMP, click here for additional guidance for information to include with your IRB application.

If relying on an independent agent or committee for DSMB services, it is the PI's responsibility to establish the services with the agent or committee. Please be reminded that the PI must submit DSMB reports to the IRB via modification or continuing review.

I. Process to monitor the progress of research and the safety of participants:

A. Monitors of the research:
The research will be monitored by several collaborators/coinvestigators involved with this investigation.
Kyle Flack, PhD, RD: Responsible for overall monitoring and reporting of all adverse events. Supervision of all assessments and obtaining informed consent. Responsible for recruitment of subjects, data collection, organization, and analyses.
Jody L. Clasey, PhD, FACSM: Serving as project mentor, aiding in oversight and direction.
Scott Black, MD: Responsible for medical oversight.
Jalyn Mason, BS, RD; Stephanie Daniel, BS; Michael Hays, BA: Responsible for recruitment of subjects, conducting assessments (resting metabolic rate, BodPod, RRV, dietary intake), and assisting in tests conducted at CCTS.

B. Frequency of data examination: To ensure the accuracy and integrity of the body composition, metabolic rate, dietary intake, and RRV data, a careful review of the data will be conducted immediately following collection procedures.

C. Monitor will examine: The monitors will review all data to detect inaccuracies in collection and analyses of the data, and review procedures immediately following any adverse events if they were to occur.

D. Procedures to insure adequate feedback: All adverse events and unusual physical discomfort will be promptly reported to the medical IRB and Dr. Scott Black. Reports to the IRB will include any decisions or recommendations made by Dr. Black regarding continuation or discontinuation of participation.

2. Process for assuring compliance with requirements regarding the reporting of adverse events (AEs):

All AE's will be immediately reported to the PI (Kyle Flack, PhD, RD) and medical director (Scot Black, MD). An AE report will be immediately filed with the IRB.
3. Process for assuring that any action resulting in a temporary or permanent suspension of research is reported to the investigator and IRB as deemed appropriate:
If any action results in a temporary or permanent suspension of the research protocol then the principal investigator will be contacted immediately. After consultation with the principal investigator the IRB will be notified so that appropriate action may take place.
4. Process for assuring data accuracy and protocol compliance:
All instrumentation will be calibrated the day of testing for each individual or group of individuals. A member of the research team will review the data to ensure data integrity and protocol adherence immediately following data collection.

5. Ensuring Confidentiality of subject's

All subject information will be locked in a file cabinet if not currently in use. Subject identification numbers specific than names, will be used following the testing

Back to Top

Subject Complaints: Describe procedures (other than information provided in consent document) for handling subject complaints or requests for information about the research. The procedures should offer a safe, confidential, and reliable channel for current, prospective, or past research subjects (or their designated representative) permitting them to discuss problems, concerns and questions, or obtain information.

Subject complaints will be collected and reported to the IRB immediately at the Office of Research Integrity at the University of Kentucky between the business hours of 8am and 5pm EST, Mon-Fri at 859-257-9428 or toll free at 1-866-400-9428.

Does your research involve **Non-English Speaking Subjects or Subjects from a Foreign Culture**?

○ Yes ◉ No

Non-English Speaking Subjects or Subjects from a Foreign Culture

Describe how information about the study will be communicated to potential subjects appropriate for their culture, and if necessary, how new information about the research may be relayed to subjects during the study.

Include contact information for someone who can act as a cultural consultant for your study. The person should be familiar with the culture of the subject population and/or be able to verify that translated documents are the equivalent of the English version of documents submitted. The consultant should not have any direct involvement with the study. If you do not know someone who would be willing to act as your cultural consultant, the Office of Research Integrity will try to find someone to fill this role (this may delay the approval process for your protocol). Please include the name, address, telephone number, and email of the person who will act as the cultural consultant for your study. For more details, see the IRB Application Instructions on Research Involving Non-English Speaking Subjects or Subjects from a Foreign Culture.

For recruitment of Non-English speaking subjects, the consent document needs to be in the subject's native language. Download the informed consent template available in the E-IRB "Informed Consent/Assent Process" section and use it as a guide for developing the consent document. (Note: Your translated consent document can be attached to your application in the "Informed Consent" section; **be sure to save your responses in this section first**.)

If research is to be conducted at an international location, identify local regulations, laws, or ethics review requirements for human subject protection. If the project has been or will be reviewed by a local Ethics Committee, attach a copy of the review to the UK IRB using the attachment button below. You may also consult the current edition of the International Compilation of Human Research Standards

Does your study involve **HIV/AIDS research and/or screening for other reportable diseases (e.g., Hepatitis**

○ Yes ◉ No

HIV/AIDS Research

If you have questions about what constitutes a reportable disease and/or condition in the state of Kentucky, see ORI's summary sheet: "Reporting Requirements for Diseases and Conditions in Kentucky" [PDF].

HIV/AIDS Research: There are additional IRB requirements for designing and implementing the research and for obtaining informed consent. Describe additional safeguards to minimize risk to subjects in the space provided below.

For additional information, visit the online IRB Survival Handbook to download a copy of the "Medical IRB's requirements for Protection of Human Subjects in Research Involving HIV Testing" [D65.0000] [PDF], and visit the Office for Human Research Protections web site for statements on AIDS research, or contact the Office of Research Integrity at 859-257-9428.

PI-Sponsored FDA-Regulated Research

Is this an investigator-initiated study that:

Back to Top

1) involves testing a Nonsignificant Risk (NSR) Device, or
2) is being conducted under an investigator-held Investigational New Drug (IND) or Investigational Device Exemption (IDE)?

○ Yes ◉ No

PI-Sponsored FDA-Regulated Research

If the answer above is yes, then the PI assumes the regulatory responsibilities of both the investigator and sponsor. The Office of Research Integrity provides a summary list of sponsor IND regulatory requirements for drug trials [PDF], IDE regulatory requirements for SR device trials [PDF], and abbreviated regulatory requirements for NSR device trials [PDF]. For detailed descriptions see FDA Responsibilities for Device Study Sponsors or FDA Responsibilities for IND Drug Study Sponsor-Investigators.

- Describe your (the PI's) experience/knowledge/training (if any) in serving as a sponsor (e.g., previously held an IND/IDE); and
- Indicate if you have transferred any sponsor obligations to a commercial sponsor, contract research organization (CRO), contract monitor, or other entity (provide details or attach FDA 1571).

IRB policy requires mandatory training for all investigators who are also FDA-regulated sponsors (see Sponsor-Investigator FAQs). A sponsor-investigator must complete the applicable Office of Research Integrity web based training, (drug or device) before final IRB approval is granted.

Has the PI completed the mandatory PI-sponsor training prior to this submission?

○ Yes ○ No

If you (the PI) have completed equivalent sponsor-investigator training, you may submit documentation of the content for the IRB's consideration.

Attachments

45819
CLOSED

HIPAA

Is HIPAA applicable? (•) Yes ( ) No

(Visit ORI's Health Insurance Portability and Accountability Act (HIPAA) web page to determine if your research falls under the HIPAA Privacy Regulation.)

If yes, check below all that apply and attach the applicable document(s):

- [ ] HIPAA De-identification Certification Form
- [x] HIPAA Waiver of Authorization

Attachments

Attach Type	File Name
CombinedInformedConsent	IRB Form C_exercise_and_weight_control_clean.pdf

STUDY DRUG INFORMATION

○ Yes ◉ No

If yes, complete the questions below. Additional study drug guidance.

LIST EACH DRUG INVOLVED IN STUDY IN THE SPACE BELOW

Drug Name:

Note: Inpatient studies are required by Hospital Policy to utilize the Investigational Drug Service (IDS). Use of IDS is highly recommended, but optional for outpatient studies. Outpatient studies not using IDS services are subject to periodic inspection by the IDS for compliance with drug accountability good clinical practices.

Indicate where study drug(s) will be housed and managed:

☐ Investigational Drug Service (IDS) UK Hospital

Other Location:

Is the study being conducted under a valid Investigational New Drug (IND) application?

○ Yes ○ No

If Yes, list IND #(s) and complete the following:

IND Submitted/Held by:

Sponsor: ☐	Held By:
Investigator: ☐	Held By:
Other: ☐	Held By:

☐ Checkmark this if the study is being conducted under FDA's Expanded Access Program (e.g., Treatment IND).

- FDA's Expanded Access Program Information (e.g., treatment IND)
- Guidance and definitions: "Expanded Access SOP" (PDF).

Please also complete and attach the Study Drug Form (PDF) (required):

Attachments

STUDY DEVICE INFORMATION

A DEVICE may be a:

- component, part, accessory;
- assay, reagent, or in-vitro diagnostic device;
- software, digital health, or mobile medical app;
- other instrument if intended to affect the structure or function of the body, diagnose, cure, mitigate, treat or prevent disease; or
- a homemade device developed by an investigator or other non-commercial entity and not approved for marketing by FDA.

For additional information, helpful resources, and definitions, see ORI's Use of Any Device Being Tested in Research web page.

Does this protocol involve testing (collecting safety or efficacy data) of a medical device including an FDA approved device, unapproved use of an approved device, humanitarian use device, and/or an investigational device?

○ Yes ● No

[Note: If a marketed device(s) is only being used to elicit or measure a physiologic response or clinical outcome, AND, NO data will be collected on or about the device itself, you may answer “no” above, save and exit this section, (Examples: a chemo drug study uses an MRI to measure tumor growth but does NOT assess how effective the MRI is at making the measurement; an exercise study uses a heart monitor to measure athletic performance but no safety or efficacy information will be collected about the device itself, nor will the data collected be used for comparative purposes against any other similar device).]

If you answered yes above, please complete the following questions.

LIST EACH DEVICE BEING TESTED IN STUDY IN THE SPACE BELOW

Device Name:

Is the study being conducted under a valid Investigational Device Exemption (IDE) or Humanitarian Device Exemption (HDE) application? See UK HUD SOP (PDF) for guidance.

○ Yes ○ No

If Yes, list IDE or HDE #(s) and complete the following:

IDE/HDE Submitted/Held by:

Sponsor: ☐ Held By:

Investigator: ☐ Held By:

Other: ☐ Held By:

☐ Check if this is a Treatment or Compassionate Use IDE under the Food and Drug Administration (FDA) Early Expanded Access program.

- FDA's Early Expanded Access Program Information
- Guidance and definitions: "Medical Device Clinical Investigations, Compassionate Use, and Treatment IDE SOP" (PDF)

Does the intended use of any device used in this study meet the regulatory definition of Significant Risk (SR) device?

○ Yes. Device(s) as used in this study presents a potential for serious risk to the health, safety, or welfare of a subject and (1) is intended as an implant; or (2) is used in supporting or sustaining human life; or (3) is of substantial importance in diagnosing, curing, mitigating or treating disease, or otherwise prevents impairment of human health; or (4) otherwise presents a potential

for serious risk to the health, safety, or welfare of a subject.

No. All devices, as used in this study do not present a potential for serious risk to the health, safety, or welfare of subjects/participants.

Please also complete and attach the Study Device Form (PDF) (required):

Attachments

RESEARCH SITES

In order for this section to be considered complete, you must click "SAVE" after ensuring all responses are accurate.

A) Check all the applicable sites listed below at which the research will be conducted. If none apply, you do not need to check any boxes.

UK Sites

- [x] UK Classroom(s)/Lab(s)
- [ ] UK Clinics in Lexington
- [ ] UK Clinics outside of Lexington
- [ ] UK Healthcare Good Samaritan Hospital
- [x] UK Hospital

Schools/Education Institutions

- [ ] Fayette Co. School Systems *
- [ ] Other State/Regional School Systems
- [ ] Institutions of Higher Education (other than UK)

***Fayette Co. School systems, as well as other non-UK sites, have additional requirements that must be addressed. See ORI's IRB Application Instructions - Off-site Research web page for details.**

Other Medical Facilities

- [ ] Bluegrass Regional Mental Health Retardation Board
- [ ] Cardinal Hill Hospital
- [ ] Eastern State Hospital
- [ ] Norton Healthcare
- [ ] Nursing Homes
- [ ] Shriner's Children's Hospital
- [ ] Veterans Affairs Medical Center
- [ ] Other Hospitals and Med. Centers

- [ ] Correctional Facilities
- [ ] Home Health Agencies
- [ ] International Sites

List all other non-UK owned/operated locations where the research will be conducted:*

*A letter of support and local context is required from non-UK sites. See *Letters of Support and Local Context* on the IRB Application Instructions - Off-Site Research web page for more information.

Attachments

B) Is this a multi-site study for which you are the lead investigator or UK is the lead site? ( ) Yes (•) No

If **YES**, you must describe the plan for the management of reporting unanticipated problems, noncompliance, and submission of

protocol modifications and interim results from the non-UK sites in the E-IRB "Research Description" section und[illegible]

If the non-UK sites or non-UK personnel are *engaged* in the research, there are additional federal and university requirements which need to be completed for their participation, such as the establishment of a cooperative IRB review agreement with the non-UK site. Questions about the participation of non-UK sites/personnel should be discussed with the ORI staff at (859) 257-9428.

45819
CLOSED

RESEARCH ATTRIBUTES

Indicate the items below that apply to your research. Depending on the items applicable to your research, you may be required to complete additional forms or meet additional requirements. Contact the ORI (859-257-9428) if you have questions about additional requirements.

☐ Not applicable

Check All That Apply

- ☐ Academic Degree/Required Research
- ☐ Aging Research
- ☐ Alcohol Abuse Research
- ☐ Cancer Research
- ☐ Certificate of Confidentiality
- ☑ CCTS-Center for Clinical & Translational Science
- ☑ Clinical Research
- ☑ Clinical Trial
- ☐ Clinical Trial Multicenter(excluding NIH Cooperative Groups)
- ☐ Clinical Trial NIH cooperative groups (i.e., SWOG, RTOG)
- ☐ Clinical Trial Placebo Controlled Trial
- ☑ Clinical Trial UK Only
- ☑ Collection of Biological Specimens
- ☐ Collection of Biological Specimens for Banking
- ☐ Community-Based Participatory Research
- ☐ Data & Safety Monitoring Board
- ☑ Data & Safety Monitoring Plan
- ☐ Deception
- ☐ Drug/Substance Abuse Research
- ☐ Educational/Student Records (e.g., GPA, test scores)
- ☐ Emergency Use (Single Patient)
- ☐ Genetic Research
- ☐ Gene Transfer
- ☐ GWAS (Genome-Wide Association Study) or NIH-funded study generating large scale genomic data
- ☐ International Research
- ☐ Internet Research
- ☐ Planned Emergency Research Involving Waiver of Informed Consent
- ☐ Pluripotent Stem Cell Research
- ☐ Recombinant DNA
- ☐ Survey Research
- ☐ Transplants
- ☐ Use of radioactive material, ionizing radiation, or x-rays [Radiation Safety Committee review required]
- ☐ Vaccine Trials

Click applicable listing(s) for additional requirements and/or information:

- Cancer Research (MCC PRMC)
- Certificate of Confidentiality (look up "Confidentiality/Privacy...")
- CCTS (Center for Clinical and Translational Science)
- Clinical Research (look up "What is the definition of....)
- Clinical Trial (look up "What is the definition of....)

Determine if research meets NIH definition of clinical trial;
*Reminder: Ensure compliance with clinicaltrials.gov registration requirements for applicable clinical trials and Good Clinical Practice (GCP) training requirements.

- Collection of Biological Specimens for Banking (look up "Specimen/Tissue Collection...")
- Collection of Biological Specimens (look up "Specimen/Tissue Collection...")
- Community-Based Participatory Research (look up "Community-Engaged...")
- Data & Safety Monitoring Board (DSMB)

*For Medical IRB: Service Request Form for CCTS DSMB

- Data & Safety Monitoring Plan
- Deception*

*For deception research, also go to the E-IRB Application Informed Consent section, checkmark and complete "Request for Waiver of Informed Consent Process"

- Emergency Use (Single Patient) [attach Emergency Use Checklist] (PDF)
- Genetic Research (look up "Specimen/Tissue Collection...")
- Gene Transfer
- HIV/AIDS Research (look up "Reportable Diseases/Conditions")
- Screening for Reportable Diseases [E2.0000] (PDF)
- International Research (look up "International & Non-English Speaking")
- NIH Genomic Data Sharing (GDS) Policy (PDF)
- Planned Emergency Research Involving Waiver of Informed Consent*

*For Planned Emergency Research Involving Waiver of Informed Consent, also go to the E-IRB Application Informed Consent section, checkmark and complete "Request for Waiver of Informed Consent Process"

- Use of radioactive material, ionizing radiation or

45819
CLOSED

x-rays for research

FUNDING/SUPPORT

If the research is being submitted to, supported by, or conducted in cooperation with an external or internal agency or funding program, indicate below all the categories that apply.

☐ Not applicable

Check All That Apply

- ☐ Grant application pending
- ☐ (HHS) Dept. of Health & Human Services
  - ☐ (NIH) National Institutes of Health
  - ☐ (CDC) Centers for Disease Control & Prevention
  - ☐ (HRSA) Health Resources and Services Administration
  - ☐ (SAMHSA) Substance Abuse and Mental Health Services Administration
- ☐ (DoJ) Department of Justice or Bureau of Prisons
- ☐ (DoE) Department of Energy
- ☐ (EPA) Environmental Protection Agency
- ☐ Federal Agencies Other Than Those Listed Here
- ☐ Industry (Other than Pharmaceutical Companies)
- ☐ Internal Grant Program w/ proposal
- ☐ Internal Grant Program w/o proposal
- ☐ National Science Foundation
- ☐ Other Institutions of Higher Education
- ☐ Pharmaceutical Company
- ☐ Private Foundation/Association
- ☐ U.S. Department of Education
- ☐ State

Other:

department start up funds

Click applicable listing(s) for additional requirements and/or information:

- (HHS) Dept. of Health & Human Services
- (NIH) National Institutes of Health
- (CDC) Centers for Disease Control & Prevention
- (HRSA) Health Resources & Services Administration
- (SAMHSA) Substance Abuse & Mental Health Services Administration
- Industry (Other than Pharmaceutical Companies) [IRB Fee Info]
- National Science Foundation
- (DoEd) U.S. Department of Education
- (DoJ) Department of Justice or Bureau of Prisons
- (DoE) Department of Energy Summary and Department of Energy Identifiable Information Compliance Checklist
- (EPA) Environmental Protection Agency

Specify the funding source and/or cooperating organization(s) (e.g., National Cancer Institute, Ford Foundation, Eli Lilly & Company, South Western Oncology Group, Bureau of Prisons, etc.):

department start up funds

Add Related Grants

If applicable, please search for and select the OSPA Account number or Electronic Internal Approval Form (eIAF) # (notif #) associated with this IRB application using the "Add Related Grants" button.
If required by your funding agency, upload your grant using the "Grant/Contract Attachments" button.

Add Related Grants

Grant/Contract Attachments

The research involves use of Department of Defense (DoD) funding, military personnel, DoD facilities, or other DoD resources.

(See DoD SOP [PDF] and DoD Summary [PDF] for details)

○ Yes ◉ No

Using the “attachments” button (below), attach applicable materials addressing the specific processes described in the DoD SOP.

DOD SOP Attachments

Additional Certification: (If your project is federally funded, your funding agency may request an Assurance/ Certification/Declaration of Exemption form.) Check the following if needed:

☐ Protection of Human Subjects Assurance/Certification/Declaration of Exemption (Formerly Optional Form – 310)

OTHER REVIEW COMMITTEES

If you check any of the below committees, additional materials may be required with your application submission.

Does your research fall under the purview of any of the other review committees listed below? [*If yes, check all that apply and attach applicable materials using the attachment button at the bottom of your screen.*]

○ Yes ◉ No

Additional Information

- ☐ Institutional Biosafety Committee
- ☐ Radiation Safety Committee
- ☐ Radioactive Drug Research Committee
- ☐ Markey Cancer Center (MCC) Protocol Review and Monitoring Committee (PRMC)
- ☐ Graduate Medical Education Committee (GME)
- ☐ Office of Medical Education (OME)

- Institutional Biosafety Committee (IBC)--Attach required IBC materials
- Radiation Safety Committee (RSC)-- For applicability, see instructions and/or upload form [WORD] [PDF]
- Radioactive Drug Research Committee (RDRC)--information
- Markey Cancer Center (MCC) Protocol Review and Monitoring Committee (PRMC)**--Attach MCC PRMC materials, if any, per instructions
- See requirement of Office of Medical Education (OME)
- See requirement of Graduate Medical Education Committee (GME)

Attachments

**** If you are proposing a study involving cancer research, be sure to have "Cancer Research" marked in the E-IRB "Research Attributes" section.** If your study involves cancer research, ORI will provide a copy of your research protocol to the Markey Cancer Center (MCC) Protocol Review and Monitoring Committee (PRMC). The MCC PRMC is responsible for determining whether the study meets the National Cancer Institute (NCI) definition of a clinical trial and for issuing documentation to you (the investigator) which confirms either that PRMC approval has been obtained or that PRC review is not required. Your IRB application will be processed and reviewed independently from the PRMC review.

ADDITIONAL INFORMATION/MATERIALS

Do you want specific information inserted into your approval letter? ○ Yes ◉ No

Approval Letter Details (e.g., serial #):

Submission Description: If you wish to have specific details included in your approval letter (e.g., serial #, internal tracking identifier, etc…), type in the box below exactly what you wish to see on the approval letter. What you type will automatically appear at the top of all approval letters, identical to how you typed it, until it is changed by you (Hint: don't include instructions or questions to ORI staff as those will appear in your approval letter). **If these details need to be changed as a result of revisions, continuation review, or modifications to the application, you are responsible for updating the content of the field below accordingly.**

Protocol/Product Attachments - For each item checked, please attach the corresponding material.

- [x] Detailed protocol
- [ ] Dept. of Health & Human Services (DHHS) approved protocol (such as NIH sponsored Cooperative Group Clinical Trial)
- [ ] Drug Documentation (e.g., Investigator Brochure; approved labeling; publication; FDA correspondence, etc.)
- [ ] Device Documentation (e.g., Manufacturer information; patient information packet; approved labeling; FDA correspondence, etc.)
- [ ] Other Documents

Protocol/Product Attachments

NOTE: Instructions for Dept. of Health & Human Services (DHHS)-approved protocol]

If you have password protected documents, that feature should be disabled prior to uploading to ensure access for IRB review.

Additional Materials:

If you have other materials you would like to include in your application for the IRB’s consideration, please attach using the Attachments button below.

[To view what materials are currently attached to your application, go to “Application Links” in the menu bar on the left and click “All Attachments”.]

Attachments

Attach Type	File Name
AdditionInfoConsiderations	EWC64_65.pdf
AdditionInfoConsiderations	180827_170758F2L_CR60_PI (00000002).pdf
AdditionInfoConsiderations	FLACK Cover Memo.pdf

SIGNATURES (ASSURANCES)

On all IRB applications there is a requirement for additional assurances by a Department Chairperson (or equivalent) [hereafter referred to as "Department Authorization" (DA)], and when applicable, a Faculty Advisor (FA) (or equivalent), which signifies the acceptance of certain responsibilities and that the science is meritorious and deserving of conduct in humans. Note: the individual assigned as DA *should not* also be listed in the Study Personnel section, the individual assigned as FA *should* be listed in the Study Personnel section.

For a list of responsibilities reflected by signing the Assurance Statement, download the guidance document "What does the Department Chairperson's Assurance Statement on the IRB application mean?"

Required Signatures:

First Name	Last Name	Role	Department	Date Signed	
Janet	Mullins	Department Authorization	Dietetics and Human Nutrition	10/31/2018 12:46 PM	View/Sign
Kyle	Flack	Principal Investigator	Dietetics and Human Nutrition	10/31/2018 10:34 AM	View/Sign

Department Authorization

☑ This is to certify that I have reviewed this research protocol and that I attest to the scientific validity and importance of this study; to the qualifications of the investigator(s) to conduct the project and their time available for the project; that facilities, equipment, and personnel are adequate to conduct the research; and that continued guidance will be provided as appropriate. When the principal investigator assumes a sponsor function, the investigator is knowledgeable of the additional regulatory requirements of the sponsor and can comply with them.

*If the Principal Investigator is also the Chairperson of the department, the Vice Chairperson or equivalent should complete the "Department Authorization".

Principal Investigator's Assurance Statement

I understand the University of Kentucky's policies concerning research involving human subjects and I agree:

1. To comply with all IRB policies, decisions, conditions, and requirements;
2. To accept responsibility for the scientific and ethical conduct of this research study;
3. To obtain prior approval from the Institutional Review Board before amending or altering the research protocol or implementing changes in the approved consent/assent form;
4. To report to the IRB in accord with IRB/IBC policy, any adverse event(s) and/or unanticipated problem(s) involving risks to subjects;
5. To complete, on request by the IRB for Full and Expedited studies, the Continuation/Final Review Forms;
6. To notify the Office of Sponsored Projects Administration (OSPA) and/or the IRB (when applicable) of the development of any financial interest not already disclosed;
7. Each individual listed as study personnel in this application has received the mandatory human research protections education (e.g., CITI);
8. Each individual listed as study personnel in this application possesses the necessary experience for conducting research activities in the role described for this research study.
9. To recognize and accept additional regulatory responsibilities if serving as both a sponsor and investigator for FDA regulated research.

☑ Furthermore, by checking this box, I also attest that:

- I have appropriate facilities and resources for conducting the study;
- I am aware of and take full responsibility for the accuracy of all materials submitted to the IRB for review;
- If applying for an exemption, I also certify that the only involvement of human subjects in this research study will be in the

45819
CLOSED

categories specified in the Protocol Type: Exemption Categories section.

- If applying for an Abbreviated Application (AA) to rely on an external IRB, I understand that certain items above (1, 3, 4, 7-8) may not apply, or may be altered due to external institutional/IRB policies. I document my agreement with the Principal Investigator Reliance Assurance Statement by digitally signing this application.

*You will be able to "sign" your assurance after you have sent your application for signatures (use Submission section). Please notify the personnel required for signing your IRB application after sending for signatures. Once all signatures have been recorded, you will need to return to this section to submit your application to ORI.

SUBMISSION INFORMATION

Each Section/Subsection in the menu on the left must have a checkmark beside it (except this Submission section) indicating the Section/Subsection has been completed; otherwise your submission for IRB review and approval will not be able to be sent to the Office of Research Integrity/IRB.

Please remember to update, when applicable, the Approval Letter Details text box under the Additional Information section to ensure verbiage you want on your approval letter is accurate.

If your materials require review at a convened IRB meeting which you will be asked to attend, it will be scheduled on the next available agenda and a message will be forthcoming to notify you of the date.

If you are making a change to an attachment, you need to delete the attachment, upload a highlighted version that contains the changes (use Document Type of "Highlighted Changes"), and a version that contains the changes without any highlights (use the appropriate Document Type for the item(s)). Do **not** delete approved attachments that are still in use.

Principal Investigator's Assurance Statement

I understand the University of Kentucky's policies concerning research involving human subjects, and I attest to:

1. Having reviewed all the investigational data from this study, including a compilation of all internal and external unanticipated problems.
2. Having reviewed, if applicable, information from the sponsor including updated investigator brochures and data and safety monitoring board reports.

I also attest that I have reviewed pertinent materials concerning the research and concluded:

- The human subject risk/benefit relationship is unaffected, mitigated, or eliminated by closure of the study and all pertinent materials for closure of the research are being submitted to the IRB for consideration.

☐ By checking this box, I am providing assurances for the applicable items listed above.

Your protocol has been submitted.

45819
CLOSED

	Document Type	File Loaded	Document Description	File Size	Mod By	Mod Date
	StudyClosure	StudyClosure.pdf		0.057	jlkear0	9/18/2019 7:47:09 AM
	CR_EntireHIPAAAuthorization	consents.pdf		0.507	kdfl224	9/5/2019 11:53:25 AM
	CombinedInformedConsent	IRB Form C_exercise_and_weight_control_clean.pdf	Clean ICF	0.234	pkma223	11/14/2018 4:05:16 PM
	AdditionInfoConsiderations	FLACK Cover Memo.pdf	CR COVER MEMO	0.133	pkma223	11/14/2018 4:04:46 PM
	AdditionInfoConsiderations	180827_170758F2L_CR60_PI (00000002).pdf	continuation form	0.124	kdfl224	11/6/2018 1:28:28 PM
	AdditionInfoConsiderations	EWC64_65.pdf	consents of last two participants	0.960	kdfl224	11/5/2018 4:09:06 PM
	DataCollection	EXERCISE REGULATIONS QUESTIONNAIRE.docx		0.013	kdfl224	7/10/2018 3:56:58 PM
	DataCollection	App14_Liking of Sedentary alt.docx		0.012	kdfl224	7/10/2018 3:56:51 PM
	DataCollection	App13_Liking of Exercise.docx		0.012	kdfl224	7/10/2018 3:56:44 PM
	DataCollection	App12_Liking of foods.docx		0.013	kdfl224	7/10/2018 3:56:38 PM
	DataCollection	App11_Food intake record.pdf		0.062	kdfl224	7/10/2018 3:56:30 PM
	DataCollection	App10_VAS scales.docx		0.034	kdfl224	7/10/2018 3:56:25 PM
	DataCollection	App8_BREVQ3.docx		0.013	kdfl224	7/10/2018 3:56:15 PM
	DataCollection	App9_vendorform.pdf		0.048	kdfl224	7/10/2018 3:55:18 PM
	DataCollection	App7_PRETTIE-Q.docx		0.013	kdfl224	7/10/2018 3:54:51 PM
	DataCollection	App6_MedhistPARQ.docx		0.014	kdfl224	7/10/2018 3:54:47 PM
	DataCollection	App5_DemographicQ.docx		0.030	kdfl224	7/10/2018 3:54:39 PM
	DataCollection	App3_Screening Application.docx		0.014	kdfl224	7/10/2018 3:54:12 PM
	Advertising	AGDHN-008_SocialMedia(PR).pdf		0.264	kdfl224	7/10/2018 3:52:48 PM
	Advertising	AGDHN-008_RM(PR).pdf		0.109	kdfl224	7/10/2018 3:52:42 PM
	Advertising	AGDHN-008_flyer(PR).pdf		1.365	kdfl224	7/10/2018 3:52:35 PM
	Advertising	AGDHN-008_monitor(PR).pdf		8.342	kdfl224	7/10/2018 3:50:37 PM

Protocol Changes

Protocol Number: 45819

No Changes

There are no recorded changes tracked for this protocol.

Study Personnel Changes:

No Changes

There are no recorded changes to study personnel.

No comments
