# Supplementary figures and images for: The consequences of exercise-induced weight loss on food reinforcement. A randomized controlled trial
Source: PLoS One. 2020 Jun 18;15(6):e0234692. doi: 10.1371/journal.pone.0234692 (PMC7302707; doi:10.1371/journal.pone.0234692)

Simple Histogram for Delta PmaxTotal

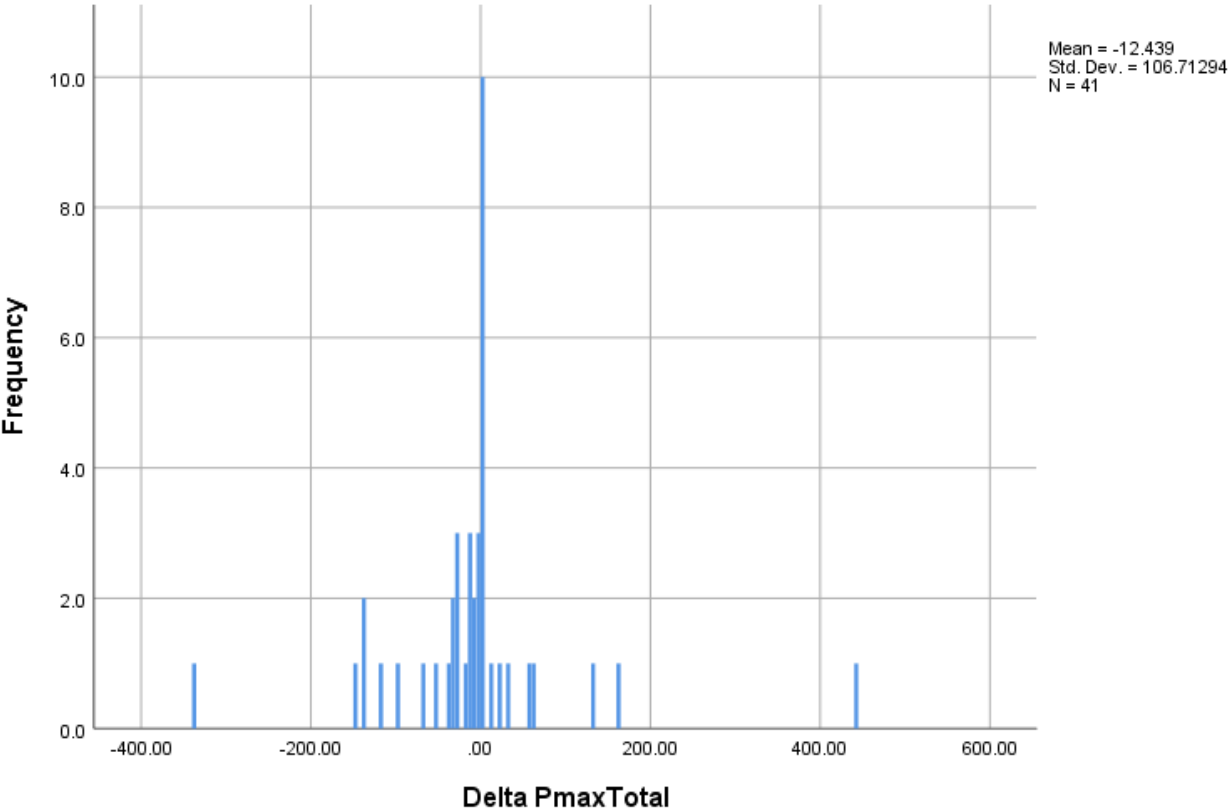

Supplement: S6 File — Histogram to depict changes in PmaxTotal. (PDF) [file pone.0234692.s007.pdf]
