# Supplementary material for: The consequences of exercise-induced weight loss on food reinforcement. A randomized controlled trial
Source: PLoS One. 2020 Jun 18;15(6):e0234692. doi: 10.1371/journal.pone.0234692 (PMC7302707; doi:10.1371/journal.pone.0234692)
Supplement: Attachment — Submitted filename: response to reviewers.pdf [file pone.0234692.s008.pdf]

Response to Reviewers

We would like to thank the reviewers and Dr. Buchowski for their time and effort into providing their important feedback. We have carefully addressed each comment and feel that this manuscript has improved significantly. Specific comments are addressed below:

1. Please explain any discrepancy between the trial protocol and the manuscript; for example, we note that, while the manuscript states that participants aged 18- 49 were included, in the registry and in the protocol the age limit is set at 40 years old.

This was incorrectly stated in the manuscript; we have now corrected this on line 91.

2. Please include additional information regarding the survey or questionnaire used in the study and ensure that you have provided sufficient details that others could replicate the analyses. For instance, if you developed a questionnaire as part of this study and it is not under a copyright more restrictive than CC-BY, please include a copy, in both the original language and English, as Supporting Information. Moreover, please include more details on how the questionnaire was pre-tested, and whether it was validated.

There were no survey/questionnaires used to gather data on primary or secondary outcomes. Participants completed questionnaires at baseline to demonstrate they were healthy enough to participate in the exercise intervention. Participants also rated their liking of the study foods at baseline and rated their hunger and desire to eat on visual analog scales prior to each RRV test. A copy of each is included as supporting information as requested.

3. Please note that according to our submission guidelines (http://journals.plos.org/plosone/s/submission-guidelines), outmoded terms and potentially stigmatizing labels should be changed to more current, acceptable terminology. For example: "Caucasian" should be changed to "white" or "of [Western] European descent" (as appropriate).

We changed "Caucasian" to "white" on line 487 and removed other potentially stigmatizing labels in this section.

4. Our internal editors have looked over your manuscript and determined that it is within the scope of our Determinants, Consequences and Management of Obesity Call for Papers. This collection of papers is headed by a team of Guest Editors for PLOS ONE:Rachel Nugent and Pratibha V. Nerurkar. Additional information can be found on our announcement page: https://collections.plos.org/s/obesity-one.
If you would like your manuscript to be considered for this collection, please let us know in your cover letter and we will ensure that your paper is treated as if you were responding to this call. If you would prefer to remove your manuscript from collection consideration, please specify this in the cover letter.

We have specified in the cover letter that we would like to include this manuscript in the Determinants, Consequences and Management of Obesity call.

5. Thank you for including the following funding information within your acknowledgements section; "The project described was supported by the NIH National Center for Advancing 410 Translational Sciences through grant number UL1TR001998"

We note that you have provided funding information that is not currently declared in your Funding Statement. However, funding information should not appear in the Acknowledgments section or other areas of your manuscript. We will only publish funding information present in the Funding Statement section of the online submission form.

Please remove any funding-related text from the manuscript and let us know how you would like to update your Funding Statement. Currently, your Funding Statement reads as follows:

"The funders had no role in study design, data collection and analysis, decision to publish, or preparation of the manuscript."

We have removed the funding-related text from the manuscript. We would like our funding statement to read: "The project described was supported by the NIH National Center for Advancing Translational Sciences through grant number UL1TR001998. The content is solely the responsibility of the authors and does not necessarily represent the official views of the NIH. The funders had no role in study design, data collection and analysis, decision to publish, or preparation of the manuscript."

6. We note that you have indicated that data from this study are available upon request. PLOS only allows data to be available upon request if there are legal or ethical restrictions on sharing data publicly. For information on unacceptable data access restrictions, please see http://journals.plos.org/plosone/s/data-availability#loc-unacceptable-data-access-restrictions.

There are no legal or ethical restrictions on sharing our data, thus we have deposited our data in a public repository and provided the DOI on Line 511 of the manuscript.

In your revised cover letter, please address the following prompts:

a) If there are ethical or legal restrictions on sharing a de-identified data set, please explain them in detail (e.g., data contain potentially identifying or sensitive patient information) and who has imposed them (e.g., an ethics committee). Please also provide contact information

for a data access committee, ethics committee, or other institutional body to which data requests may be sent.

b) If there are no restrictions, please upload the minimal anonymized data set necessary to replicate your study findings as either Supporting Information files or to a stable, public repository and provide us with the relevant URLs, DOIs, or accession numbers. Please see http://www.bmj.com/content/340/bmj.c181.long for guidelines on how to de-identify and prepare clinical data for publication. For a list of acceptable repositories, please see http://journals.plos.org/plosone/s/data-availability#loc-recommended-repositories.

We will update your Data Availability statement on your behalf to reflect the information you provide.

7.Please upload a copy of Figure 1, to which you refer in your text on page 13. If the figure is no longer to be included as part of the submission please remove all reference to it within the text.

Our figure has been uploaded; we apologize for overlooking this in the initial submission.

8. Please include captions for your Supporting Information files at the end of your manuscript, and update any in-text citations to match accordingly. Please see our Supporting Information guidelines for more information: http://journals.plos.org/plosone/s/supporting-information.

We have included captions for our supporting information as requested at the end of our manuscript, lines 674-682

Reviewer #1: Overall
This paper builds upon both theoretical considerations, the impact of physical activity upon dietary reinforcement, while also offering practical advice for those interested in losing weight via physical activity, maintain lean mass via resistance training. The overall paper is well written and adds to the literature. The additions I would suggest are as follows:
Introduction:
Line 40: add that exercise also essential to maintaining healthy weight after loss. Considerable research evidence supports this.

We certainly agree with this statement and included "and weight loss maintenance" in this statement as our reference (#5) also backs this (line 42).

Line 62: "seek rewards FOR exercising …"

This has been edited as suggested, now line 63.

Methods:

Line 84: Can you say "increase food reinforcement" if measuring 2 food types?

This is a fine point and we further clarified that we hypothesized food reinforcement for specifically high energy dense foods available would increase (line 85-86).

Line 146: Did any participants report an atypical diet (keto, Atkins etc.) diet or that they were currently dieting to lose weight at baseline?

Participants were not dieting to lose weight at baseline. This was an inclusion criterion, along with being weight stable. We realize this was not included in the participant characteristics section and now include both of these criteria (line 101-102)

Line: 191 spell-check of "unhealthy"

This has been corrected, now line 210.

Line 186-191: Pmax can be calculated for healthy and unhealthy separately. Specify that Pmax_total is for both added together. Also, why did you only examine Pmax total and not the separate Pmaxs for each food group?

The last two sentences of this section state "P_{max} healthy and P_{max} unhealthy were added together, reflecting the total number of schedules completed for food, or P_{max}Total. Thus, P_{max}Total reflects an overall score of total food reinforcement, irrespective of type (healthy or unhealthy)" There were no differences in changes in P_{max} healthy or P_{max} unhealthy, we specified this in Table 3, results (line 284), and touched on it in the discussion (lines 409-412).

Mention that participants' favorite food (healthy or unhealthy) may not have been offered and they would respond differently to different options

We have included this point when addressing limitations of the present study, lines 484-486..

Did you examine proportion of food earned during the RRV task to food consumed afterwards? Those results may be interesting.

There were only two occasions where participants did not consume all the food they earned in the RRV task, thus these analyses were not preformed. We have stated this more specifically on line 202.

Results
Line 237-238: Because changes in body composition are so important for study results, please include body fat percentage, and kg of fat mass and lean mass in Tables 2.

We have now included baseline values for body fat %, FM and FFM in table 2.

Line 264: Unhealthy food, to keep terminology consistent

This change has been made, now line 285.

Discussion:
RRV task and DXA took place very shortly after end of a very intense training regime. How might results change if participants were given opportunity to taper and/or rest before post-intervention results?

This is an interesting question and we have expanded on this regarding when RRV testing is done in relation to the exercise intervention, lines 456-461

Any indication whether or not participants change their diets, either intentionally or unintentionally?

We have included, lines 470-475, a couple sentences about how we believe they did not intentionally engage in restrictive eating behaviors but likely increased their food intake in response to the exercise intervention.

Reviewer #2: This is a well-crafted study that examines a novel and important topic. I enjoyed reading it. I have listed some suggestions, by section, below. While I believe you can handle these issues relatively easily, I do think they will strengthen the manuscript and should be addressed. If these issues are addressed, I believe the manuscript warrants publication. Well done!

Introduction

“The most prevalent mechanism responsible for maintaining energy homeostasis during an exercise program is increases in energy intake, largely due to the fact that the rate of energy intake far exceeds the rate of energy expenditure” while I agree with this point, it may be worthwhile to discuss metabolic changes that can occur with weight loss. At least mention adaptive thermogenesis as a possible mechanism complicating weight loss from exercise. It’s possible that alterations in food reinforcement may be part of the adaptive thermogenesis response.

We certainly agree with this point and have mentioned physiologic adaptations to an exercise-induced energy deficit that also work to return the body back to energy balance, lines 46-48)

Methods

"… 52 completed all baseline tests and were randomized into one of three groups…" I think it's a good idea to list the three groups parenthetically here. Revie to "… 52 completed all baseline tests and were randomized into one of three groups (six exercise sessions per week, two weekly sessions, sedentary control)…"

We have made this addition as suggested, line 93.

"The study was a randomized, controlled trial that included a 12-week exercise intervention of either six sessions (days) per week, two sessions per week, or a sedentary control group (no exercise) blocked on gender" list the number of males and females in each group.

We have included this as suggested on lines 126-127

How was compliance of the sedentary group assessed?

We have included in our 481-484 that we did not assess the physical activity of the control group during the intervention but believe they remained sedentary as their body weight did not change. We included this in the section on the weaknesses of the study.

What were some of the exercise options available to your exercise groups? Did they only do cardio (e.g., treadmill, bike) or were they able to do resistance training?

Participants only engaged in aerobic exercise, this is now detailed on lines 135-138

Why was liking only assessed at baseline? Changes in hedonics as a result of the intervention would have been interesting to look at as well as RRV.

We agree this would have been interesting to include and noted this in the limitations section, lines 462-464.

Interesting uses of Pmax for the RRV task. I like how you quantified RRV but, out of curiosity, why not just use raw Pmax scores for healthy versus unhealthy foods as your measure of RRV?

We combined the Pmax scores to get an overall food reinforcement score, not relative to anything. But we were also interested if they may increase their reinforcing value of one type of food relative to the other, so maybe they wouldn't change Pmax of healthy or unhealthy independently, but when put against each other, we may see a significant change in the

proportion (maybe people find candy bars more reinforcing than dried banana after exercising). We were worried that assessing the reinforcing value of food relative to a non-food activity (reading or watching TV for example) may have been skewed as people may prefer doing a sedentary activity opposed to exercise if they were burnt out from the intervention. Thus, this would have been more of a test for the reinforcing value of sedentary activities than food and confounded our results. In response to reviewer 1, we provided the results on changes in Pmax for healthy and unhealthy foods in Table 3, the results section, and provided a bit on the discussion on this (lines 409-412)

"Change scores were also tested if significantly different from zero via T-tests." Were these done for each group separately?

These were done for each group separately, now indicated on line 263-264..

"Differences in changes in…" revise to "Differences in change scores (post value minus baseline value) in…"

This has been revised as suggested, lines 476-477

Discussion

"The present findings, demonstrating FFM deficits are the root cause in the increase…" this language is too definitive for my liking. Revise to "The present findings, demonstrating FFM deficits were the root cause in the increase…" or something similar.

We have revised as suggested, line 439.

"Our findings indicate that there is great variability in individuals' change in food reinforcement after a 12-week aerobic exercise intervention, and those who do increase their food reinforcement are those who lose the greatest amount of FFM post-intervention…" Again this reads as too definitive. I think it's important to describe the results only within the context of the present study. Stating "those who do increase their food reinforcement are those who lose the greatest amount of FFM post-intervention" sounds like you are concluding this is what happens with weight loss for everyone. While that may be true, you cannot conclude that from this single study. I know this seems like a small detail, but it is important to remain tentative. Revise to "Our findings indicate that there was great variability in individuals' change in food reinforcement after a 12-week aerobic exercise intervention, and those who did increase their food reinforcement were also those who lost the greatest amount of FFM post-intervention…" This way you are only talking about your results.

We agree with this statement and have made the changes to the conclusion as suggested, line 495.
