# Supplementary material for: The consequences of exercise-induced weight loss on food reinforcement. A randomized controlled trial
Source: PLoS One. 2020 Jun 18;15(6):e0234692. doi: 10.1371/journal.pone.0234692 (PMC7302707; doi:10.1371/journal.pone.0234692)
Supplement: Attachment — Submitted filename: response to reviewers2.docx [file pone.0234692.s010.docx]

Response to Reviewers

We would like to thank the reviewers and Dr. Buchowski for their time and effort into providing their important feedback. We have carefully addressed each comment and feel that this manuscript has improved significantly. Since we satisfied the previous reviewers’ comments, we are responding below only to Reviewer #3’s comments, with our response in italics below each comment.

**COMMENTS – Manuscript PONE-D-20-01440R1**

Title: “The consequences of exercise-induced weight loss on food reinforcement. A randomized controlled trial”

**Important note: This review pertains only to ‘statistical aspects’ of the study and so** **‘****clinical aspects’ [like medical importance, relevance of the study,** **‘clinical significance and implication(s)’ of the whole study, etc.] are to be evaluated [should be assessed] separately/independently.**

My first question is when title says “A randomized controlled trial” then why this study is classified as [Article Type:] ‘Research Article’? If it is really ‘A randomized controlled trial’ then it is expected to estimate ‘required sample size’. For the study on this topic a small effect size is generally assumed. Even for medium effect size [type I error 5%, Power=80%] according to ‘table-2 on page 158 of an old but classical paper “A power primer” in Psychological Bulletin, 1992, vol.:112, pp 155-159 the required sample size is 52 per group. However, in this study total sample size is only 52. [For small effect size (type I error 5%, Power=80%) it is as large as 322]. Note that in lines 498-500 it is said that “it appears a large sample size is required to better elucidate food reinforcement changes after exercise, potentially taking sex effects into account”. But a priori estimation of the required sample size is not found.

*Thank you for pointing this out, I initially selected ‘Research Article’ as this is what I believed a manuscript of this nature to fall under, although this study does fit NIH’s description of a clinical trial, so this may need to be updated. I have contacted the journal for advice in changing this designation.*

*As for the second part of this question, I’m not certain I am understanding this fully. I have provided a power analysis, starting on line 270, and have added detail regarding how and why the study is powered this way. In short, this study is powered to detect a significant difference in body fat loss between groups. Although not specific to the outcome measures of the present investigation, powering the study in this manner allows us the ability to assess the present outcomes in groups that differ in body fat loss, an important, clinically relevant, variable to consider.*

What exactly you want to convey by ‘blocked on gender’ [a sedentary control group (no exercise) blocked on gender], the phrase used (in line 123) while describing group-III? {If that means ‘matched’ why the wide differences (68.4%, 85%, 78.8%, in Gr.I,II,III respectively)? It cannot be usual meaning of ‘block’ because group sizes are so different (19, 20, 14 in Gr.I,II,III respectively)}. Please explain.

*You are correct, this wasn’t “blocked” in the traditional sense. Rather, men and women were randomized separately. This has been updated and explained on lines 124/125.*

According to line 260 “Group differences in baseline study characteristics where tested via 1-way ANOVA” but P-values are not reported in table-2 displayed in lines 505- {which is in fact very good as it is often said that

To provide a description of baseline characteristics is entirely reasonable (since it is clearly important in assessing to whom the results of the trial can be applied), however, it does not require the division of baseline characteristics by treatment groups. Statistical comparison of baseline characteristics is not desirable at all [because even if P-value turns out to be significant (while comparing baseline characteristics despite random allocation), it is, by definition, a false positive] as you then are supposed to be testing ‘randomization’ then, which in any single trial may not balance all baseline characteristics because ‘randomization’ is a sort of ‘insurance’ and not a guarantee scheme}.

*I do know many statisticians who scoff at the notion of testing for bassline group differences in a randomized trial. Although I’ve also received comments on papers I have submitted where reviewers ask me to test for baseline group differences despite a randomized design. I’m not a statistician, I have no preference, and have therefore removed the mention of testing for group differences (line 260) and simply state that baseline data is presented in table 2 (line 282).*

If the study was to observe effect of ‘progressive ratio’, then why [as said in lines 148-9] ‘Participants in the two-day per week group were instructed to perform two long exercise sessions per week and encouraged to try to expend 1,000 kcal per session’ is not understood. Will you please thro light on purpose [of that action]. ‘Power Analysis’ account given in lines 271 onwards is not very convincing though ‘was based on our previous study’.

*The progressive ration (or PR schedules) refers to the food reinforcement assessment. The two and six days of exercise per week were the exercise intervention. We were testing to see if the intervention exerted an effect on the food reinforcement measure (PR schedules). To make this clearer, we have added a couple lines at the end of the I intro, lines 87-89. As noted in the prior comment, we have added detail to the power analysis.*

In Table 3 [Resulting data from the 12-week exercise intervention between groups that exercised either six or two days per week and a sedentary control group] data are mean ± SE whereas “It may please be noted that ‘data always should be presented as means ± standard deviation’ [and never as means ± standard errors]. The standard deviation (SD) and standard error (SE) of the mean measures two very different things. The standard error depends heavily upon the sample size. [Note: standard error of the mean tells not about variability in the original population, as the standard deviation does, but about the certainty with which a sample mean estimates the true population mean. Since the certainty with which we can estimate the mean increases as the sample size increases, the standard error of the mean decreases as the sample size increases.] People are interested in knowing variability in ‘study population’ and not precision in estimate.

*We understand this notion of this perspective and the difference in SD and SE, which is why Table 1 included SD, as we are reporting strictly on our sample in this table. I have always been advised to report SE when presenting results from a clinical trial as we are trying to present our data not just specific to our data set but to convey it to the larger population. Although again, if SD is preferred in this scenario we are fine with making these changes. Table 3 has been updated to reflect SD.*

It is definitely appreciable that [line 294-297] ‘Quantile regression analysis’ [predicting changes in PmaxTotal is presented in Table 5: controlling for weekly exercise energy expenditure, exercise frequency per week (group), and changes in FM, REE and hunger] is used, however, a brief note (at least its non-parametric nature) on this ‘not so popular’ technique was desirable (my opinion may be little bias as a biostatistician). Surprisingly, median or other quartiles are not reported {though this non-parametric technique is used}. As you may be aware, there are disadvantages as well as limitations [like ‘monotonicity constraints’ as pointed out in ‘Journal of Machine Learning Research Non-parametric Quantile Estimation (2005)’ or computations are quite tedious compared to the least squares method] which is why technique, though very old, is seldom used. I believe [not sure] that “quantile regression” (because said in lines 270 that “All analyses were performed) is available in IBM SPSS Version 26.

*We appreciate the insight, and to better report all quartiles, we have included a histogram for delta P_max_Total and have included this in the supplemental files and referenced it on line 292.*

Limitation mentioned in lines 462-64 [Liking of the test foods was not assessed as baseline liking scores did not influence food reinforcement, but if may have been interesting to see how or if liking of these tests foods changed as a result of the exercise intervention] is questionable. In the backdrop of the clear mention in lines 475-6 that this study is secondary of a larger study, [“the analysis, being a secondary aim of a larger study, was likely underpowered to detect significant differences in change scores”], editors may think about publication of such an article {though other things presented are alright}.

*It is true that this was a secondary aim of a larger study, but the analysis was centered on the regression analysis, identifying predictors in food reinforcement. Just because there were no statistically significant changes in P_max_, the regression analysis was still able to demonstrate significant findings. With changes in P_max_Total over +20 in the 6-d group and -39 and -14 in the 2-d and control groups, respectively, this seems like a fair speculation and worthy of publication, albeit probably not in Nature or N Engl J Med. This notion that liking may have changed during the intervention to influence food reinforcement at post-testing was brought up by another reviewer before, questioning if liking was assessed at 12-weeks and if there were changes in this measure. Since we did not assess liking at 12 weeks, we considered this a limitation.*
